# Stereoselective synthesis of perillaldehyde-based chiral β-amino acid derivatives through conjugate addition of lithium amides

**DOI:** 10.3762/bjoc.10.289

**Published:** 2014-11-21

**Authors:** Zsolt Szakonyi, Reijo Sillanpää, Ferenc Fülöp

**Affiliations:** 1 Institute of Pharmaceutical Chemistry, University of Szeged, H-6720 Szeged, Eötvös utca 6, Hungary; 2 Department of Chemistry, University of Jyväskylä, POB 35, 40351 Jyväskylä, Finland; 3 Stereochemistry Research Group of the Hungarian Academy of Sciences, H-6720 Szeged, Eötvös u. 6, Hungary

**Keywords:** asymmetric synthesis, β-amino acid, chiral, Michael addition, monoterpene

## Abstract

The Michael addition of dibenzylamine to (+)-*tert*-butyl perillate (**3**) and to (+)-*tert*-butyl phellandrate (**6**), derived from (*S*)-(−)-perillaldehyde (**1**), resulted in diastereomeric β-amino esters **7A**–**D** in a moderately stereospecific reaction in a ratio of 76:17:6:1. After separation of the diastereoisomers, the major product, cis isomer **7A**, was quantitatively isomerized to the minor component, *trans*-amino ester **7D**. All four isomers were transformed to the corresponding β-amino acids **10A**–**D**, which are promising building blocks for the synthesis of β-peptides and 1,3-heterocycles in three steps. The steric effects of the isopropyl group at position 4 and of the α-methyl substituent of (*R*)-*N*-benzyl-*N*-α-methylbenzylamine on the reactivity were also studied and, upon application of a chiral amine, excellent stereoselectivity of the conjugate addition was observed. Amino ester **11** was obtained as a single product and transformed to the corresponding amino acids **10A** and **10D** in good yields on the gram scale.

## Introduction

In the past decade, cyclic β-amino acids proved to be versatile building blocks both in pharmacological developments and asymmetric syntheses [1–8]. Alicyclic and bicyclic chiral β-amino acids have played a key role in the synthesis of β-peptide-type foldamers, where through the selection of an appropriate alicyclic or bicyclic ring system, the backbone stereochemistry, stereochemical patterning or additional functional groups, well-defined β-helical (e.g., β-H12, β-H14, β-H16 or β-H18) or β-sheet structures can be prepared [9–13]. While it is primarily the backbone stereochemistry that determines the secondary structure of foldamers, the introduction of well-designed hydrophilic or hydrophobic substituents on the alicyclic ring of β-amino acids can modify the fine structure of β-peptides.

There are several powerful synthetic methods through which alicyclic or bicyclic β-amino acid enantiomers can be obtained. These include the selective reduction of β-enamino ester enantiomers [14], enzyme-catalyzed kinetic resolution [15], and a variety of asymmetric syntheses, for example, the enantioselective syntheses of β-lactams followed by ring opening [16–17], or the enantioselective desymmetrization of achiral anhydrides followed by Curtius degradation [18–20].

The highly stereoselective Michael addition of lithium amide-type nucleophiles to α,β-unsaturated esters also proved to be a very efficient and useful method for the preparation of alicyclic β-amino acids in homochiral form [21–22]. Generally, in these transformations, the source of chirality is served by chiral lithium amides, and there are only few examples where chiral α,β-unsaturated esters are applied [23–27].

Easily obtainable chiral monoterpenes, such as (+)-3-carene as well as all the enantiomers of pulegone, α-pinene and verbenone, have frequently been used as starting materials for the preparation of chiral reagents and as unique synthons in asymmetric syntheses of β-amino acids and 1,3-amino alcohols, which in turn can be applied as chiral additives, catalysts or building blocks [17,28–34]. From this aspect, chiral, monoterpene-based α,β-unsaturated esters might be excellent starting materials, in which the natural monoterpene skeleton may serve as the chiral origin for the stereoselective construction of the β-amino acid moiety.

Our present aim was the synthesis of new, limonene-based chiral β-amino acid derivatives derived from commercially available (−)-perillaldehyde (**1**). These 4-isopropyl-substituted analogues of ACHC (2-aminocyclohexanecarboxylic acid) might serve as promising building blocks for the synthesis of chiral 1,3-heterocycles and foldamers [7,11,23,35].

## Results and Discussion

**Scheme 1:**
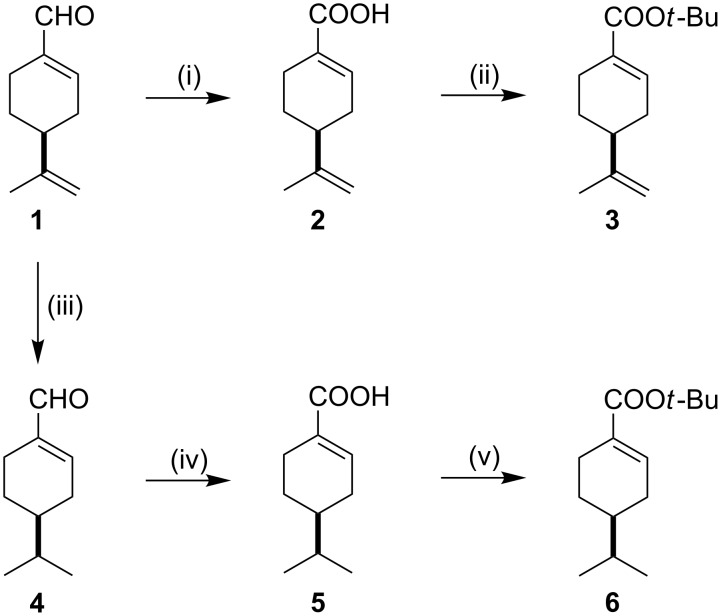
Reagents and conditions: (i) 2-methyl-2-butene, *t*-BuOH, NaClO_2_ (aq), NaH_2_PO_4_ (aq), yield: 60%; (ii) (CF_3_CO)_2_O, dry toluene, *t*-BuOH, rt, yield: 53%; (iii) 5% Pt/C, 1 atm H_2_, *n*-hexane/EtOAc 1:1, 12 h, rt, yield: 77%; (iv) 2-methyl-2-butene, *t*-BuOH, NaClO_2_ (aq), NaH_2_PO_4_ (aq), yield: 58%; (v) (CF_3_CO)_2_O, dry toluene, *t*-BuOH, rt, yield: 48%.

The key intermediate Michael acceptor, *tert*-butyl perillate (**3**), was prepared by a combination of literature protocols, starting from commercially available (−)-(4*S*)-perillaldehyde (**1**) in a two-step reaction. First, oxidation of **1** led to perillic acid (**2**) [36], which was subsequently converted to the *tert*-butyl ester (**3**) [37]. In order to study the steric effect of the more bulky isopropyl group on the Michael addition, (4*S*)-*tert*-butyl phellandrate (**6**) was prepared via (4*S*)-phellandral (**4**) and (4*S*)-phellandric acid (**5**) (Scheme 1) [38–40].

**Scheme 2:**
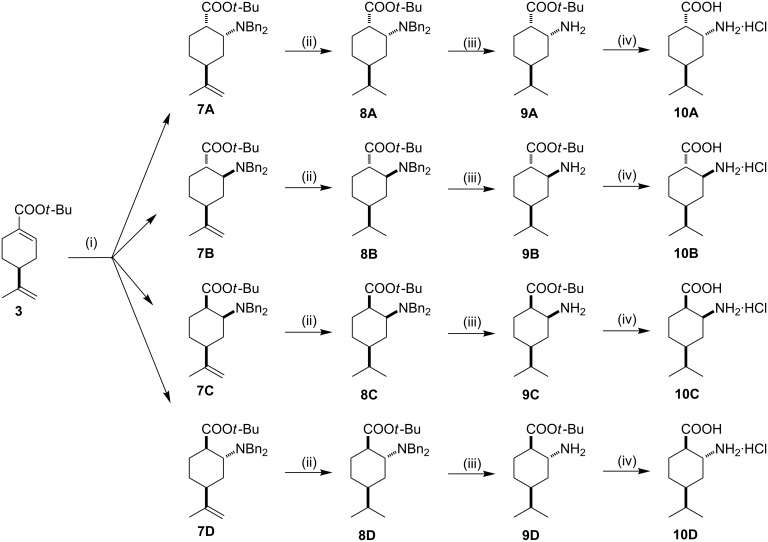
Reagents and conditions: (i) 2.4 equiv LiNBn_2_, dry THF, −78 °C, 6 h, then NH_4_Cl (aq), overall yield: 87% (isomeric mixture), ratio **7A**:**7B**:**7C**:**7D** = 76:17:6:1; (ii) 5% Pt/C, *n*-hexane/EtOAc 1:1, 1 atm. H_2_, rt, 16 h, yield: 90–92%; (iii) 5% Pd/C, *n*-hexane/EtOAc 1:1, 1 atm H_2_, rt, 24 h, yield: 92–95%; (iv) 10% HCl (aq), rt, 24 h, yield: 90–94%.

**Figure 1:**
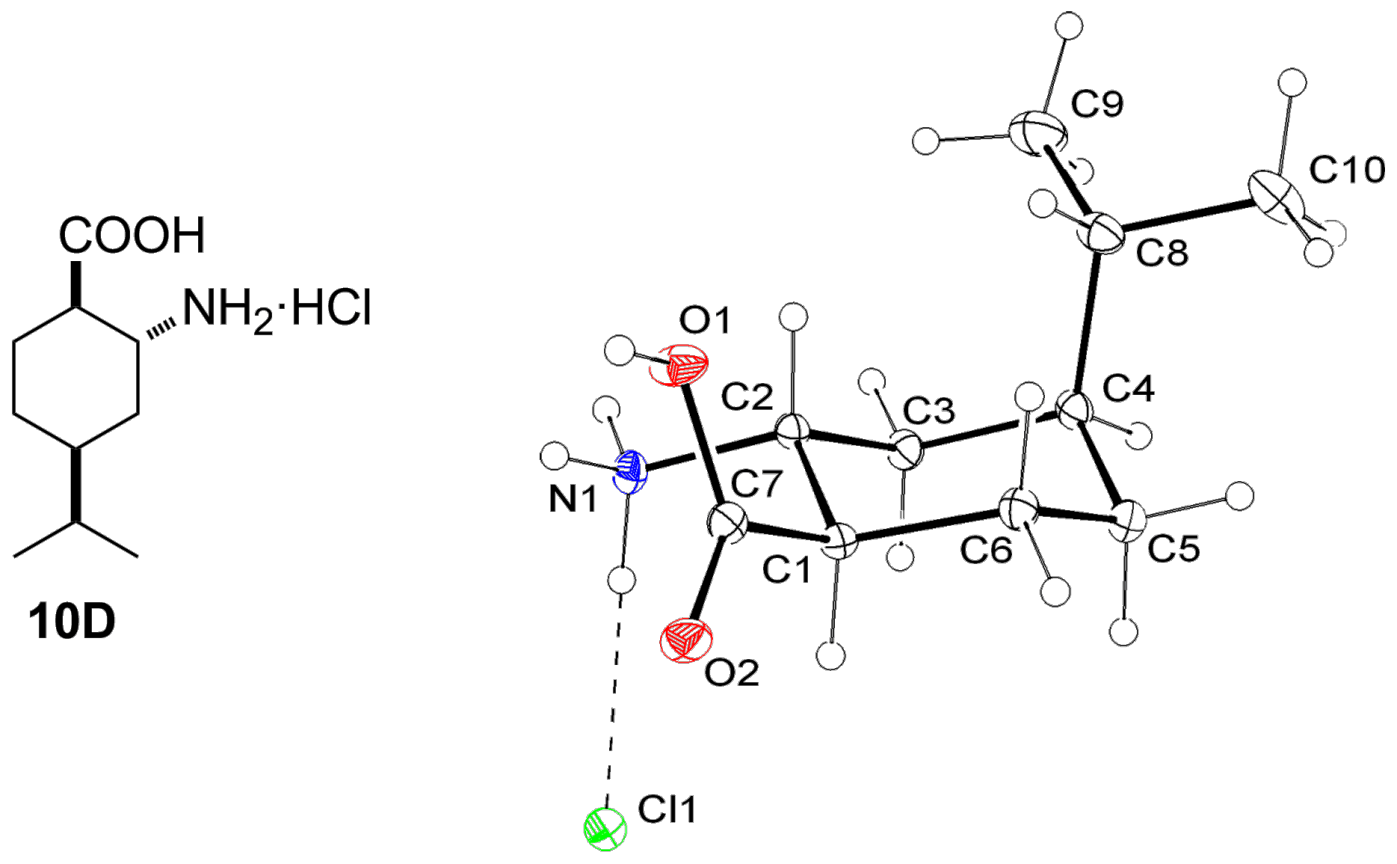
Structure of **10D** and an ORTEP plot of its configuration.

The asymmetric Michael addition was accomplished by the reaction of in situ generated achiral lithium dibenzylamide with compound **3** following a published protocol [23], to exploit the effect of the isopropenyl group on the cyclohexene ring. An NMR study of the crude product demonstrated the good stereoselectivity of the addition. The ^1^H NMR measurements of the crude product indicated that all four possible diastereosomers are formed in a ratio **7A**:**7B**:**7C**:**7D** = 76:17:6:1 (Scheme 2). The diastereoisomers **7A**–**D** could be successfully separated through a two-step chromatographic process, and their relative configurations were determined by 2D NMR techniques. Remarkable nOe correlations were observed between C2-H and C9-Me (**10A** and **10D**), between C1-H and C8-H (**10A**), and a weak effect was found between C1-H and C8-H (**10B**) (see Figure 1 for numbering).

Amino esters **7A**–**D** were transformed to the appropriate amino acids **10A**–**D** in three steps. The selective reduction of the isopropenyl double bond over a Pt/C catalyst resulted in **8A**–**D**. The subsequent removal of the benzyl groups by hydrogenolysis over palladium on carbon (Pd/C) in a 1:1 mixture of *n*-hexane/EtOAc for 24 h gave primary amino esters **9A**–**D** in excellent yields. The final hydrolysis of the ester groups under acidic conditions successfully led to amino acids **10A**–**D**.

In addition to the NOESY experiments, the relative configuration of **10D** was determined by means of X-ray crystallography (Figure 1).

**Scheme 3:**
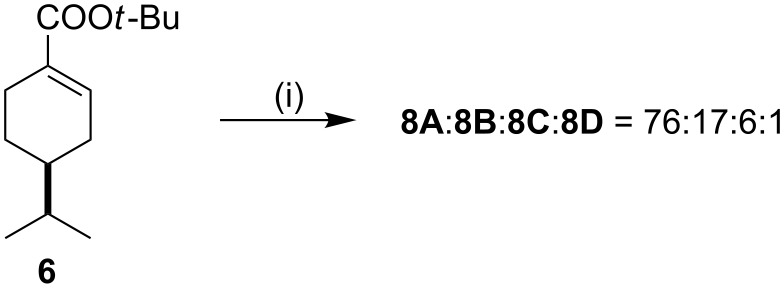
Reagents and conditions: (i) 2.4 equiv LiNBn_2_, dry THF, −78 °C, 6 h, then NH_4_Cl (aq), overall yield: 85% (isomeric mixture).

The Michael addition was also carried out on **6**, the 7,8-dihydro analogue of *tert*-butyl perillate (**3**), however the saturation of the isopropenyl function at position 4 proved to have no effect on the stereoselectivity of the reaction (Scheme 3).

**Scheme 4:**
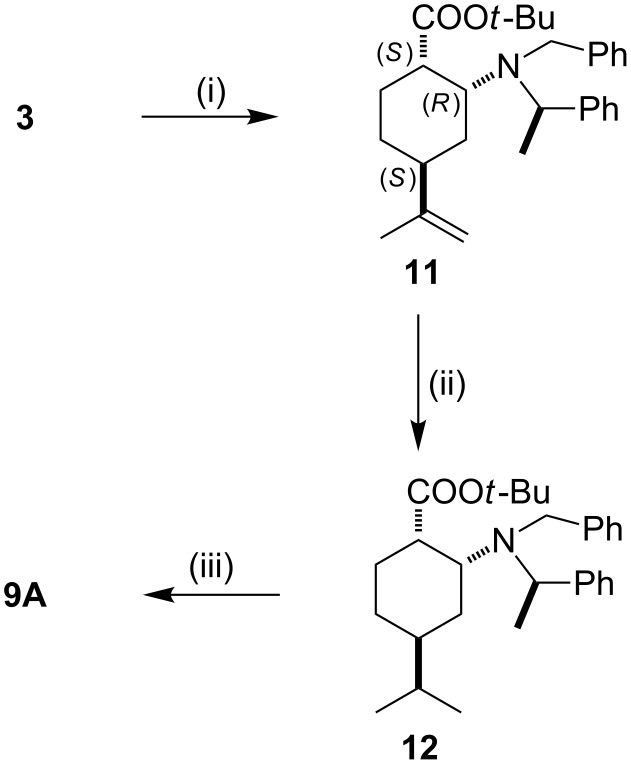
Reagents and conditions: (i) 2.4 equiv lithium (*R*)-*N*-benzyl-*N*-α-methylbenzylamide, dry THF, −78 °C, 6 h, then NH_4_Cl (aq), yield: 88%; (ii) 5% Pt/C, *n*-hexane/EtOAc 1:1, 1 atm H_2_, rt, 16 h, yield: 91%; (iii) 5% Pd/C, *n*-hexane/EtOAc 1:1, 1 atm H_2_, rt, 16 h, yield: 90%.

When *N*-benzyl-*N*-α-methylbenzylamine was applied as a chiral nucleophile in the conjugate addition, the steric effect of the α-methyl substituent could be investigated. The addition was proven highly stereoselective (de > 99%), based on the ^1^H NMR data of the crude product and *cis*-amino ester **11** as a single product was obtained in gram-scale quantities and high yield (Scheme 4). In addition to the NOESY examinations, the relative stereochemistry of **11** was also proven through its conversion to **9A** in two steps.

Applying (*S*)-*N*-benzyl-*N*-α-methylbenzylamide as a chiral lithium amide, only formation of the mixture of diastereoisomers with very low yield (ca. 10%) was observed.

**Scheme 5:**
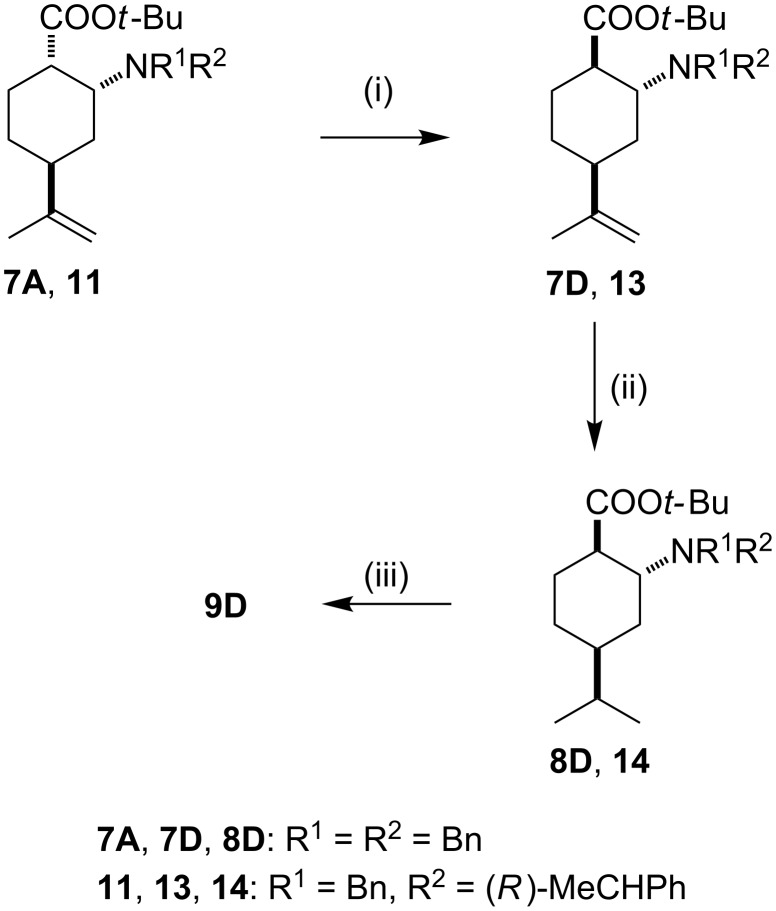
Reagents and conditions: (i) 0.2 equiv KO*t*-Bu/*t*-BuOH, 40 °C, 24 h, yield: 93% (**7D**), 91% (**13**); (ii) 5% Pt/C, *n*-hexane/EtOAc 1:1, 1 atm H_2_, rt, 16 h, yield: 90% (**8D**), 91% (**14**); (iii) 5% Pd/C, *n*-hexane/EtOAc 1:1, 1 atm H_2_, rt, 16 h, yield: 92%.

Under alkaline conditions, *cis*-amino esters **7A** and **11** underwent isomerization at the carboxylic function, resulting in *trans*-amino esters **7D** and **13** in excellent yields (Scheme 5). The relative stereochemistry of **13** was proven through its conversion to **9D** in two steps. This rapid and quantitative isomerization allows the gram-scale synthesis of the minor component amino acid **10D** (see Scheme 2).

## Conclusion

In conclusion, the highly stereoselective Michael addition of lithium dibenzylamide and (*R*)-*N*-benzyl-*N*-α-methylbenzylamide to *tert*-butyl perillate (**3**) proved to be an efficient method for the preparation of limonene-based β-amino acids through the three-step transformation of the resulting *N*,*N*-dialkyl β-amino esters **7A**–**D** and **11**. The minor component, *trans*-amino acid **10D**, was successfully prepared on gram-scale quantities through the facile isomerization of the *cis-*amino esters under alkaline conditions. It appears likely that the resulting new monomers **10A**–**D** incorporated in a β-peptide sequence will be able to force the formation of unique β-helix or β-sheet structures, thereby affording a novel route to promising β-peptides.

## Supporting Information

File 1 General information, experimental details, characterization data and copies of ^1^H and ^13^C NMR spectra.
