# Supplementary material for: Stereoselective synthesis of perillaldehyde-based chiral β-amino acid derivatives through conjugate addition of lithium amides
Source: Beilstein J Org Chem. 2014 Nov 21;10:2738–42. doi: 10.3762/bjoc.10.289 (PMC4273274; doi:10.3762/bjoc.10.289)

Supporting information

for

Stereoselective synthesis of perillaldehyde-based chiral β-amino acid derivatives through conjugate addition of lithium amides

Zsolt Szakonyi[1], Reijo Sillanpää[2], Ferenc Fülöp[1,3,$]*

Address: [1]Institute of Pharmaceutical Chemistry, University of Szeged, H-6720 Szeged, Eötvös utca 6, Hungary, [2]Department of Chemistry, University of Jyväskylä, POB 35, 40351 Jyväskylä, Finland and [3]Stereochemistry Research Group of the Hungarian Academy of Sciences, H-6720 Szeged, Eötvös u. 6, Hungary

Email: fulop@pharm.u-szeged.hu

*Corresponding author

[$]Tel.: +36-62-545564; Fax: +36-62-545705

General information, experimental details, characterization data and copies of ^{1}H and ^{13}C NMR spectra

Contents

Preparation and characterization data of compounds **6**–14 .... S3-S14

References S14

^{1}H, ^{13}C NMR and HMBC spectra of new compounds S15–S37

General informations

^{1}H NMR spectra were recorded on a Bruker Avance DRX 400 spectrometer at 400.13 MHz (^{1}H) and 100.61 MHz (^{13}C) [δ = 0 (TMS)] in $CDCl_3$, DMSO-d_6 or D_2O in a 5-mm tube. Chemical shifts are expressed in ppm (δ) relative to TMS as internal reference. *J* values are given in Hz. Microanalyses were performed on a Perkin-Elmer 2400 elemental analyser. Optical rotations were obtained with a Perkin-Elmer 341 polarimeter. Melting points were determined on a Kofler apparatus and are uncorrected. Chromatographic separations were carried out on Merck Kieselgel 60 (230-400 mesh ASTM). Reactions were monitored with Merck Kieselgel 60 F_{254}-precoated tlc plates (0.25 mm thickness).

(–)-(4*S*)-Perillaldehyde and (+)-(1*R*)-*N*-benzylphenylethylamine are commercially available. THF and toluene were dried over Na wire; all other chemicals and solvents were used as supplied. (–)-(4*S*)-Perillic acid and (4*S*)-4-isopropylcyclohex-1-enecarboxylic acid ((4*S*)-phellandric acid) were prepared by literature methods, and were identical with those reported therein [1–4].

Experimental details

(–)-(4*S*)-*tert*-Butyl perillate (3)

To a solution of (–)-perillic acid (7.20 g, 43.3 mmol) in dry toluene (60 mL), trifluoroacetic anhydride (16.4 mL, 24.47 g, 116.4 mmol) was added at room temperature. The resulting homogeneous solution was stirred for 40 min and then treated with *t*BuOH (52.0 g, 696 mmol) with ice-bath cooling. The solution was stirred for 4 h at room temperature, and the mixture was then diluted with toluene (200 mL), cooled to 0 °C and extracted, first with 10% aqueous NaOH solution (100 mL), then with H_2O (100 mL) and finally with brine (100 mL). The organic phase was dried (Na_2SO_4), filtered and concentrated *in vacuo*. The crude product was purified by column chromatography on silica gel (*n*-hexane–Et_2O = 14:1), resulting in compound **3** as a colourless oil (5.10 g, 53%); $[\alpha]_D^{20}$ = –86.0 (*c* = 0.25, MeOH); δ_H (400 MHz, $CDCl_3$) 1.38–1.47 (1H, *m*), 1.48 (9H, *s*), 1.74 (3H, *s*), 1.83–1.91 (1H, *m*), 2.02–2.24 (3H, *m*), 2.26–2.35 (1H, *m*), 2.39–2.48 (1H, *m*), 4.69–4.73 (1H, *m*), 4.74–4.76 (1H, *m*),

6.86–6.91 (1H, *m*); δ_C (100 MHz, $CDCl_3$) 21.1, 25.1, 27.6, 28.5, 31.5, 40.6, 80.1, 109.4, 131.5, 138.1, 148.9, 166.7. Anal. Calcd. for $C_{14}H_{24}O_2$ (222.32): C, 75.63; H, 9.97; Found: C, 75.90; H, 10.19.

(4*S*)-*tert*-Butyl 4-isopropylcyclohex-1-enecarboxylate (6)

Starting from **5** (3.64 g, 21.6 mmol), the synthesis of **6** was accomplished analogously as prescribed for **3**, furnishing unsaturated ester **6** as a colourless oil (2.33 g (48%); $[\alpha]_D^{20}$ = –96.0 (*c* 0.25 in MeOH); δ_H (400 MHz, $CDCl_3$) 0.93 (3H, *d*, *J* = 6.6 Hz), 0.94 (3H, *d*, *J* = 6.6 Hz), 1.22 (1H, *ddd*, *J* = 5.1, 11.5, 23.8 Hz), 1.27–1.38 (1H, *m*), 1.48–1.58 (1H, *m*), 1.52 (9H, *s*), 1.82–1.97 (2H, *m*), 2.08–2.31 (2H, *m*), 2.41–2.50 (1H, *m*), 6.88–6.92 (1H, *m*); δ_C (100 MHz, $CDCl_3$) 20.0, 20.2, 25.3, 26.2, 28.6, 29.8, 32.4, 39.7, 80.1, 132.2, 138.8, 167.4. Anal. Calcd. for $C_{14}H_{24}O_2$ (224.34): C, 74.95; H, 10.78; Found: C, 75.24; H, 10.87.

Syntheses of amino esters 7A–D and 11: general procedure 1

n-BuLi solution (27 mL of a 1.6 M solution in *n*-hexane) was added dropwise to a stirred solution of secondary amine (45.1 mmol) in dry THF at –78 °C under an argon atmosphere, followed by stirring for 30 min prior to the addition of acceptor **3** or **6** (18.0 mmol) in dry THF (25 mL) at –78 °C. After the appropriate reaction time (6 h), saturated aqueous NH_4Cl solution (100 mL) was added and the solution was warmed to room temperature, partitioned between Et_2O (3x200 mL) and brine, dried (Na_2SO_4) and concentrated *in vacuo*. Purification by column chromatography gave the desired products (the diastereomeric ratio of the crude product was determined by ^1H NMR measurement to be **7A**:**7B**:**7C**:**7D** = 76:17:6:1; **11** was obtained as a single diastereoisomer).

(1*S*,2*R*,4*S*)-*tert*-Butyl 2-dibenzylamino-4-isopropenylcyclohexane-1-carboxylate (7A)

According to *General procedure 1*, **7A** was prepared from **3** with dibenzylamine. Purification was accomplished by column chromatography on silica gel: the mixture of **7A** and **7B** was first separated from the mixture of **7C** and **7D** with *n*-hexane–Et_2O = 19:1; repeated chromatography with a mixture of *n*-hexane–Et_2O–AcOH = 94:5:1 then resulted in isolated compound **7A** (4.99 g, 66.1%). An oil;

$[\alpha]_D^{20}$ = +15.0 (*c* 0.25 in MeOH); δ_H (400 MHz, $CDCl_3$) 1.33–1.54 (2H, *m*), 1.44 (9H, *s*), 1.67 (3H, *s*), 1.71–1.89 (2H, *m*), 1.93–2.02 (1H, *m*), 2.30–2.38 (1H, *m*), 2.44–2.52 (1H, *m*), 2.60 (1H, *dt*, *J* = 4.8, 8.9 Hz), 3.33 (1H, *dt*, *J* = 4.6, 6.3 Hz), 3.67 (2H, *d*, *J* = 13.9 Hz), 3.78 (2H, *d*, *J* = 13.9 Hz), 4.66 (2H, *d*, *J* = 25.4 Hz), 7.16–7.36 (10H, *m*); δ_C (100 MHz, $CDCl_3$) 21.9, 24.9, 28.0, 28.5, 30.3, 39.9, 46.7, 54.7, 55.5, 80.4, 109.7, 127.0, 128.4, 129.3, 140.5, 149.0, 174.6. Anal. Calcd. for $C_{28}H_{37}NO_2$ (419.60): C, 80.15; H, 8.89; N, 3.34; Found: C, 80.23; H, 9.06; N, 3.11.

(1*S*,2*S*,4*S*)-*tert*-Butyl 2-dibenzylamino-4-isopropenylcyclohexane-1-carboxylate (7B)

According to *General procedure 1*, **7B** was prepared from **3** with dibenzylamine. Purification was accomplished by column chromatography on silica gel: the mixture of **7A** and **7B** was first separated from the mixture of **7C** and **7D** with *n*-hexane–Et_2O = 19:1; repeated chromatography with a mixture of *n*-hexane–Et_2O–AcOH = 94:5:1 then resulted in isolated compound **7B** (1.12 g, 14.8%). An oil; $[\alpha]_D^{20}$ = –8.0 (*c* 0.255 in MeOH); δ_H (400 MHz, $CDCl_3$) 1.38–1.49 (2H, *m*), 1.46 (9H, *s*), 1.66–1.77 (2H, *m*), 1.73 (3H, *s*), 1.80–1.93 (2H, *m*), 2.23 (1H, *dd*, *J* = 12.0, 24.1 Hz), 2.70–2.82 (1H, *m*), 2.84–2.93 (1H, *m*), 3.74 (4H, *br s*), 4.70 (2H, *d*, *J* = 8.8 Hz), 7.14–7.42 (10H, *m*); δ_C (100 MHz, $CDCl_3$) 21.0, 26.8, 28.5, 29.0, 29.4, 43.2, 46.0, 55.0, 60.3, 80.1, 109.0, 127.0, 128.5, 128.8, 140.6, 149.9, 174.8. Anal. Calcd. for $C_{28}H_{37}NO_2$ (419.60): C, 80.15; H, 8.89; N, 3.34; Found: C, 80.31; H, 9.07; N, 3.20.

(1*R*,2*S*,4*S*)-*tert*-Butyl 2-dibenzylamino-4-isopropenylcyclohexane-1-carboxylate (7C)

According to *General procedure 1*, **7C** was prepared from **3** with dibenzylamine. Purification was accomplished by column chromatography on silica gel: the mixture of **7C** and **7D** was first separated from the mixture of **7A** and **7B** with *n*-hexane–Et_2O = 19:1; repeated chromatography with a mixture of *n*-hexane–Et_2O–AcOH = 94:5:1 then resulted in isolated compound **7C** (0.39 g, 5.2%). An oil; $[\alpha]_D^{20}$ = +64.0 (*c* 0.255 in MeOH); δ_H (400 MHz, $CDCl_3$) 1.04–1.23 (2H, *m*), 1.41–1.50 (1H, *m*), 1.47 (9H, *s*), 1.67–1.76 (1H, *m*), 1.72 (3H, *s*), 1.81–1.95 (2H, *m*), 1.97–2.01 (1H, *m*), 2.47 (1H, *dt*, *J* = 3.7, 11.6 Hz), 2.95 (1H, *dt*, *J* = 3.4, 11.6 Hz), 3.42 (2H, *d*, *J* = 13.5 Hz), 3.82 (2H, *d*, *J* = 13.5 Hz), 4.68–

4.72 (2H, *m*), 7.17–7.34 (10H, *m*); δ_C (100 MHz, $CDCl_3$) 21.3, 28.5, 29.5, 30.0, 30.8, 44.5, 49.4, 54.1, 59.5, 80.1, 108.9, 127.1, 128.3, 129.5, 140.3, 150.1, 174.9. Anal. Calcd. for $C_{28}H_{37}NO_2$ (419.60): C, 80.15; H, 8.89; N, 3.34; Found: C, 80.39; H, 9.09; N, 3.14.

(1*R*,2*R*,4*S*)-*tert*-Butyl 2-dibenzylamino-4-isopropenylcyclohexane-1-carboxylate (7D)

Method A: According to *General procedure 1*, **7D** was prepared from **3** with dibenzylamine. Purification was accomplished by column chromatography on silica gel: the mixture of **7C** and **7D** was first separated from the mixture of **7A** and **7B** with *n*-hexane–Et_2O = 19:1; repeated chromatography with *n*-hexane–Et_2O–AcOH = 94:5:1 then resulted in isolated compound **7D** (66 mg, 0.9%).

Method B: According to *General procedure 3*, **7D** was prepared from **7A** (6.12 g, 93%).

An oil; $[\alpha]_D^{20}$ = –32.0 (*c* 0.25 in MeOH); δ_H (400 MHz, $CDCl_3$) 1.35–1.60 (4H, *m*), 1.47 (9H, *s*), 1.62–1.71 (1H, *m*), 1.64 (3H, *s*), 1.86–1.94 (1H, *m*), 2.42–2.47 (1H, *m*), 2.51 (1H, *dt*, *J* = 4.2, 11.0 Hz), 3.13 (1H, *dt*, *J* = 3.4, 11.7 Hz), 3.46 (2H, *d*, *J* = 13.6 Hz), 3.82 (2H, *d*, *J* = 13.6 Hz), 4.59 (1H, *s*), 4.73–4.76 (1H, *m*), 7.15–7.34 (10H, *m*); δ_C (100 MHz, $CDCl_3$) 22.9, 26.0, 27.0, 27.5, 28.5, 39.2, 49.5, 54.1, 54.7, 80.0, 111.5, 127.1, 128.2, 129.5, 140.5, 146.1, 175.0. Anal. Calcd. for $C_{28}H_{37}NO_2$ (419.60): C, 80.15; H, 8.89; N, 3.34; Found: C, 80.37; H, 9.11; N, 3.10.

(1*S*,2*R*,4*S*)-*tert*-Butyl 2-[benzyl-(1-(1*R'*)-phenylethyl)-amino]-4-isopropenyl-1-cyclohexanecarboxylate (11)

According to *General procedure 1*, **11** was prepared from **3** and (1*R*)-phenylethylamine. Purification was accomplished by column chromatography on silica gel (*n*-hexane–Et_2O = 19:1), resulting in compound **11** (7.10 g, 88%) as an oil; $[\alpha]_D^{20}$ = +93.0 (*c* 0.25 in MeOH); δ_H (400 MHz, $CDCl_3$) 1.29 (3H, *d*, *J* = 6.8 Hz), 1.41–1.59 (4H, *m*), 1.45 (9H, *s*), 1.70 (3H, *s*), 1.78–1.94 (2H, *m*), 2.36–2.50 (3H, *m*), 3.04–3.11 (1H, *m*), 3.94 (2H, *dd*, *J* = 15.0, 27.7 Hz), 4.07 (1H, *dd*, *J* = 6.8, 13.5 Hz), 4.67 (1H, *s*), 4.79 (1H, *s*), 7.14–7.44 (10H, *m*); δ_C (100 MHz, $CDCl_3$) 16.7, 22.8, 24.4, 25.3, 28.5, 29.6, 40.0, 46.1, 51.4, 54.0, 58.2, 80.2, 110.7, 126.6, 126.8, 128.1, 128.2, 128.3, 128.4, 143.2, 145.0, 147.5, 175.3. Anal. Calcd. for $C_{29}H_{39}NO_2$ (433.63): C, 80.33; H, 9.07; N, 3.23; Found: C, 80.47; H, 9.18; N, 3.01.

(1*R*,2*R*,4*S*)-*tert*-Butyl 2-[benzyl-(1-(1*R'*)-phenylethyl)-amino]-4-isopropenyl-1-cyclohexanecarboxylate (13): General procedure 3

To a solution of **11** (7.0 g, 15.68 mmol) in *t*BuOH (150 mL), KO*t*Bu (0.69 g, 6.15 mmol) was added and the solution was stirred at 40 °C for 24 h. The solution was evaporated to one-tenth volume, diluted with Et_2O (200 mL) and extracted with ice-cold water (3x150 mL). The organic phase was dried (Na_2SO_4) and concentrated *in vacuo*. The crude product was purified by column chromatography on silica gel (*n*-hexane–Et_2O = 19:1), resulting in compound **13** (6.39 g, 91%); an oil; $[\alpha]_D^{20}$ = –13.0 (*c* 0.25 in MeOH); δ_H (400 MHz, $CDCl_3$) 1.25–1.38 (2H, *m*), 1.36 (3H, *d*, *J* = 6.8 Hz), 1.46 (9H, *s*), 1.50–1.72 (3H, *m*), 1.62 (3H, *s*), 1.75–1.83 (1H, *m*), 1.94–2.02 (1H, *m*), 2.32 (1H, *dt*, *J* = 4.5, 9.9 Hz), 2.36–2.42 (1H, *m*), 3.29 (1H, *dt*, *J* = 3.5, 10.7 Hz), 3.71 (1H, *d*, *J* = 14.8 Hz), 3.85 (1H, *d*, *J* = 14.8 Hz), 4.12 (1H, *dd*, *J* = 6.8, 13.9 Hz), 4.67 (1H, *s*), 4.61 (1H, *s*), 4.75–4.78 (1H, *m*), 7.13–7.35 (10H, *m*); δ_C (100 MHz, $CDCl_3$) 18.7, 22.7, 25.9, 27.4, 28.5, 31.0, 39.6, 49.4, 50.4, 55.9, 59.3, 79.9, 111.1, 126.7, 126.8, 128.1, 128.2, 128.5, 128.8, 142.7, 145.4, 146.8, 175.2. Calcd. for $C_{29}H_{39}NO_2$ (433.63): C, 80.33; H, 9.07; N, 3.23; Found: C, 80.51; H, 9.21; N, 2.97.

Syntheses of amino esters 8A–D, 12 and 14: general procedure 2

To a suspension of platinum-on-carbon (5% Pt/C, 0.180 g) in a 1:1 mixture of *n*-hexane–EtOAc (160 mL), the appropriate amino ester **7A–D**, **11** or **13** (3.15 mmol) in a 1:1 mixture of *n*-hexane–EtOAc (10 mL) was added, and the resulting mixture was stirred under a H_2 atmosphere (1 atm) at room temperature for 16 h. The suspension was filtered through a Celite pad and the solvent was evaporated off. The oily crude product obtained was purified by column chromatography (silica gel, *n*-hexane–Et_2O = 19:1), affording a colourless oily product.

(1*S*,2*R*,4*S*)-*tert*-Butyl 2-dibenzylamino-4-isopropylcyclohexane-1-carboxylate (8A)

According to *General procedure 2*, **8A** was prepared from **7A** (1.28 g, 91%); an oil; $[\alpha]_D^{20}$ = –35.0 (*c* 0.125 in MeOH); δ_H (400 MHz, $CDCl_3$) 0.82 (3H, *d*, *J* = 6.5 Hz), 0.84 (3H, *d*, *J* = 6.5 Hz), 1.13–1.24

(1H, *m*), 1.30–1.41 (2H, *m*), 1.44 (9H, *s*), 1.47–1.56 (1H, *m*), 1.64–1.92 (3H, *m*), 2.18–2.27 (1H, *m*); 2.58 (1H, *dt*, *J* = 5.0, 9.6 Hz); 3.25 (1H, *dt*, *J* = 4.5, 7.1 Hz); 3.64 (2H, *d*, *J* = 13.9 Hz), 3.78 (2H, *d*, *J* = 13.9 Hz), 7.15–7.36 (10H, *m*); δ_C (100 MHz, $CDCl_3$) 20.7, 20.8, 24.8, 26.5, 28.5, 28.8, 30.2, 40.0, 46.8, 54.6, 55.6, 80.3, 127.0, 128.4, 129.2, 140.7, 174.8. Anal. Calcd. for $C_{28}H_{39}NO_2$ (421.61): C, 79.76; H, 9.32; N, 3.32; Found: C, 79.93; H, 9.55; N, 3.01.

(1*S*,2*S*,4*S*)-*tert*-Butyl 2-dibenzylamino-4-isopropylcyclohexane-1-carboxylate (8B)

According to *General procedure 2*, **8B** was prepared from **7B** (1.25 g, 90%); an oil; $[\alpha]_D^{20}$ = –4.0 (*c* 0.125 in MeOH); δ_H (400 MHz, $CDCl_3$) 0.87 (3H, *d*, *J* = 6.5 Hz), 0.88 (3H, *d*, *J* = 6.5 Hz), 0.98–1.10 (1H, *m*), 1.26–1.52 (4H, *m*), 1.45 (9H, *s*), 1.62–1.69 (1H, *m*), 1.77–1.85 (1H, *m*), 1.95 (1H, *dd*, *J* = 12.2, 24.4 Hz); 2.63–2.73 (1H, *m*); 2.83–2.92 (1H, *m*), 3.73 (4H, *dd*, *J* = 14.6, 17.7 Hz), 7.15–7.40 (10H, *m*); δ_C (100 MHz, $CDCl_3$) 20.1, 20.3, 24.6, 28.3, 28.5, 29.4, 33.3, 43.5, 44.6, 55.0, 60.6, 80.3, 126.9, 128.4, 128.8, 141.2, 175.3. Anal. Calcd. for $C_{28}H_{39}NO_2$ (421.61): C, 79.76; H, 9.32; N, 3.32; Found: C, 79.89; H, 9.69; N, 3.21.

(1*R*,2*S*,4*S*)-*tert*-Butyl 2-dibenzylamino-4-isopropylcyclohexane-1-carboxylate (8C)

According to *General procedure 2*, **8C** was prepared from **7C** (1.29 g, 92%); an oil; $[\alpha]_D^{20}$ = +51.0 (*c* 0.25 in MeOH); δ_H (400 MHz, $CDCl_3$) 0.80–0.85 (1H, *m*), 0.85 (3H, *d*, *J* = 2.3 Hz), 0.87 (3H, *d*, *J* = 2.3 Hz), 0.93 (1H, *dd*, *J* = 12.0, 23.7 Hz); 0.99–1.09 (1H, *m*), 1.34–1.47 (2H, *m*), 1.46 (9H, *s*), 1.60–1.68 (1H, *m*), 1.84–1.95 (2H, *m*), 2.41 (1H, *dt*, *J* = 3.8, 11.7 Hz); 2.88 (1H, *dt*, *J* = 3.6, 11.6 Hz); 3.42 (2H, *d*, *J* = 13.5 Hz), 3.81 (2H, *d*, *J* = 13.5 Hz), 7.16–7.33 (10H, *m*); δ_C (100 MHz, $CDCl_3$) 20.0, 20.1, 27.6, 28.5, 28.8, 30.1, 33.1, 43.4, 49.8, 54.1, 59.9, 79.9, 127.0, 128.2, 129.6, 140.4, 175.1. Anal. Calcd. for $C_{28}H_{39}NO_2$ (421.61): C, 79.76; H, 9.32; N, 3.32; Found: C, 79.85; H, 9.47; N, 3.19.

(1*R*,2*R*,4*S*)-*tert*-Butyl 2-dibenzylamino-4-isopropylcyclohexane-1-carboxylate (8D)

According to *General procedure 2*, **8D** was prepared from **7D** (1.25 g, 90%); an oil; $[\alpha]_D^{20}$ = –46.0 (*c* 0.125 in MeOH); δ_H (400 MHz, $CDCl_3$) 0.81 (3H, *d*, *J* = 6.3 Hz), 0.86 (3H, *d*, *J* = 6.3 Hz), 1.20–1.41

(4H, *m*), 1.48 (9H, *s*), 1.42–1.69 (3H, *m*), 2.05–2.13 (1H, *m*), 2.49 (1H, *dt*, *J* = 4.3, 11.6 Hz); 3.02 (1H, *dt*, *J* = 3.5, 11.6 Hz); 3.40 (2H, *d*, *J* = 13.5 Hz), 3.80 (2H, *d*, *J* = 13.5 Hz), 7.15–7.35 (10H, *m*); δ_C (100 MHz, $CDCl_3$) 21.5, 21.7, 25.5, 26.2, 26.5, 27.5, 28.5, 41.1, 49.5, 54.1, 54.2, 80.0, 127.1, 128.2, 129.5, 140.6, 174.6. Anal. Calcd. for $C_{28}H_{39}NO_2$ (421.61): C, 79.76; H, 9.32; N, 3.32; Found: C, 79.90; H, 9.52; N, 3.13.

(1*S*,2*R*,4*S*)-*tert*-Butyl 2-[benzyl-(1-(1*R'*)-phenylethyl)-amino]-4-isopropyl-1-cyclohexanecarboxylate (12)

According to *General procedure 2*, **12** was prepared from **11** (1.28 g, 91%); an oil; $[\alpha]_D^{20}$ = +66.0 (*c* 0.255 in MeOH); δ_H (400 MHz, $CDCl_3$) 0.80 (3H, *d*, *J* = 6.7 Hz), 0.83–0.89 (1H, *m*), 0.91 (3H, *d*, *J* = 6.6 Hz), 1.24–1.30 (1H, *m*), 1.29 (3H, *d*, *J* = 6.8 Hz), 1.32–1.53 (4H, *m*), 1.45 (9H, *s*), 1.66–1.78 (2H, *m*), 1.64–1.92 (3H, *m*), 2.28 (1H, *dt*, *J* = 4.5, 12.9 Hz); 2.36–2.43 (1H, *m*); 2.99 (1H, *dt*, *J* = 4.5, 12.2 Hz); 3.88 (1H, *d*, *J* = 14.7 Hz), 3.98–4.09 (2H, *m*); 7.15–7.48 (10H, *m*); δ_C (100 MHz, $CDCl_3$) 16.0, 21.6, 21.7, 23.7, 24.8, 26.9, 28.5, 29.0, 41.5, 46.3, 51.5, 53.3, 58.0, 80.1, 126.6, 126.8, 128.1, 128.2, 128.3, 128.5, 143.3, 145.2, 175.4. Calcd. for $C_{29}H_{41}NO_2$ (435.64): C, 79.95; H, 9.49; N, 3.22; Found: C, 80.11; H, 9.69; N, 3.03.

(1*R*,2*R*,4*S*)-*tert*-Butyl 2-[benzyl-(1-(1'*R*)-phenylethyl)-amino]-4-isopropyl-1-cyclohexanecarboxylate (14)

According to *General procedure 2*, **14** was prepared from **13** (1.28 g, 91%); an oil; $[\alpha]_D^{20}$ = –29.0 (*c* 0.265 in MeOH); δ_H (400 MHz, $CDCl_3$) 0.80 (3H, *d*, *J* = 6.4 Hz), 0.81 (3H, *d*, *J* = 6.4 Hz), 1.13–1.22 (1H, *m*), 1.28–1.35 (1H, *m*), 1.37 (3H, *d*, *J* = 7.0 Hz), 1.41–1.51 (2H, *m*), 1.46 (9H, *s*), 1.53–1.71 (3H, *m*), 1.84–1.90 (1H, *m*); 2.27 (1H, *dt*, *J* = 4.3, 10.8 Hz); 3.23 (1H, *dt*, *J* = 3.3, 10.7 Hz); 3.69 (1H, *d*, *J* = 14.5 Hz), 3.81 (1H, *d*, *J* = 14.4 Hz), 4.04–4.11 (1H, *m*), 7.14–7.33 (10H, *m*); δ_C (100 MHz, $CDCl_3$) 19.0, 21.3, 21.9, 25.7, 26.7, 27.3, 28.6, 30.5, 41.3, 49.9, 50.2, 52.3, 59.6, 79.9, 126.7, 126.8, 128.1, 128.2, 128.5, 129.1, 142.6, 145.4, 175.5. Calcd. for $C_{29}H_{41}NO_2$ (435.64): C, 79.95; H, 9.49; N, 3.22; Found: C, 80.16; H, 9.61; N, 3.00.

Syntheses of amino esters 9A–D: general procedure 4

To a suspension of palladium-on-carbon (5% Pd/C, 0.55 g) in a mixture of *n*-hexane–EtOAc = 1:1 (120 mL), the appropriate amino ester (4.74 mmol) in a mixture of *n*-hexane–EtOAc = 1:1 (10 mL) was added, and the resulting mixture was stirred under a H_2 atmosphere (1 atm) at room temperature for 24 h. The suspension was filtered through a Celite pad and the solvent was removed. The oily crude product obtained was purified by column chromatography (silica gel, toluene–EtOH = 9:1), affording a colourless oily product.

(1*S*,2*R*,4*S*)-*tert*-Butyl 2-amino-4-isopropylcyclohexane-1-carboxylate (9A)

According to *General procedure 4*, **9A** was prepared from **8A** (1.08 g, 94%); an oil; $[\alpha]_D^{20}$ = –39.0 (*c* 0.125 in MeOH); δ_H (400 MHz, $CDCl_3$) 0.84 (3H, *d*, *J* = 2.5 Hz), 0.85 (3H, *d*, *J* = 2.5 Hz), 0.86–0.99 (1H, *m*), 1.21–1.42 (4H, *m*), 1.44 (9H, *s*), 1.70–1.78 (3H, *m*), 2.27 (1H, *dt*, *J* = 3.3, 12.2 Hz), 3.48–3.53 (1H, *m*); δ_C (100 MHz, $CDCl_3$) 19.9, 20.2, 22.3, 28.6, 29.0, 32.9, 36.6, 37.1, 48.3, 48.9, 80.5, 174.6. Anal. Calcd. for $C_{14}H_{27}NO_2$ (241.37): C, 69.66; H, 11.27; N, 5.80; Found: C, 69.89; H, 11.41; N, 5.60.

(1*S*,2*S*,4*S*)-*tert*-Butyl 2-amino-4-isopropylcyclohexane-1-carboxylate (9B)

According to *General procedure 4*, **9B** was prepared from **8B** (1.05 g, 92%); an oil; $[\alpha]_D^{20}$ = +19.0 (*c* 0.125 in MeOH); δ_H (400 MHz, $CDCl_3$) 0.87 (6H, *d*, *J* = 6.8 Hz), 0.99–1.20 (2H, *m*), 1.40–1.56 (4H, *m*), 1.46 (9H, *s*), 1.60–1.69 (1H, *m*), 2.08–2.15 (1H, *m*); 2.65–2.69 (1H, *m*); 2.72 (1H, *dt*, *J* = 4.5, 11.6 Hz), δ_C (100 MHz, $CDCl_3$) 20.1, 20.2, 25.1, 28.6, 29.1, 33.0, 36.2, 44.0, 47.5, 52.6, 80.6, 176.1. Anal. Calcd. for $C_{14}H_{27}NO_2$ (241.37): C, 69.66; H, 11.27; N, 5.80; Found: C, 69.83; H, 11.39; N, 5.65.

(1*R*,2*S*,4*S*)-*tert*-Butyl 2-amino-4-isopropylcyclohexane-1-carboxylate (9C)

According to *General procedure 4*, **9C** was prepared from **8C** (1.08 g, 95%); an oil; $[\alpha]_D^{20}$ = +31.0 (*c* 0.505 in MeOH); δ_H (400 MHz, $CDCl_3$) 0.87 (6H, *d*, *J* = 6.7 Hz), 0.84–0.91 (1H, *m*), 0.95 (1H, *ddd*, *J* = 3.3, 12.1, 25.3 Hz), 1.14–1.48 (3H, *m*), 1.46 (9H, *s*), 1.67–1.75 (1H, *m*), 1.81–1.99 (3H, *m*), 2.86 (1H, *ddd*, *J* = 4.0, 10.1, 13.9 Hz); δ_C (100 MHz, $CDCl_3$) 20.1, 20.2, 28.6, 28.9, 29.3, 32.9, 38.6, 43.2, 52.3, 54.5, 80.6, 175.3. Anal. Calcd. for $C_{14}H_{27}NO_2$ (241.37): C, 69.66; H, 11.27; N, 5.80; Found: C, 69.85; H, 11.47; N, 5.62.

(1*R*,2*R*,4*S*)-*tert*-Butyl 2-amino-4-isopropylcyclohexane-1-carboxylate (9D)

According to *General procedure 4*, **9D** was prepared from **8D** (1.05 g, 92%); an oil; $[\alpha]_D^{20}$ = –40.0 (*c* 0.125 in MeOH); δ_H (400 MHz, $CDCl_3$) 0.86 (3H, *d*, *J* = 6.7 Hz), 0.90 (3H, *d*, *J* = 6.7 Hz), 1.20–0.45 (4H, *m*), 1.46 (9H, *s*), 1.55–1.86 (4H, *m*), 2.07–2.16 (1H, *m*), 3.14–3.25 (1H, *m*); δ_C (100 MHz, $CDCl_3$) 21.0 (2xMe), 23.8, 27.0, 28.4, 28.6, 35.8, 39.6, 47.2, 52.1, 80.6, 174.6. Anal. Calcd. for $C_{14}H_{27}NO_2$ (241.37): C, 69.66; H, 11.27; N, 5.80; Found: C, 69.87; H, 11.40; N, 5.59.

Syntheses of amino acid hydrochlorides 10A–D: general procedure 5

The appropriate amino ester **9A–D** (2.41 g, 10 mmol) was dissolved in a mixture of Et_2O (15 mL) and 10% aqueous HCl solution (100 mL), which was followed by stirring at room temperature for 24 h. The mixture was then evaporated to dryness and the resulting white crystalline product was washed with Et_2O and filtered off.

(1*S*,2*R*,4*S*)-2-Amino-4-isopropylcyclohexanecarboxylic acid hydrochloride (10A)

According to *General procedure 5*, **10A** was obtained as white crystals (2.03 g, 92%); mp 210–213 °C; $[\alpha]_D^{20}$ = +46.0 (*c* 0.125 in MeOH); δ_H (400 MHz, DMSO–d_6) 0.83 (3H, *d*, *J* = 2.5 Hz), 0.84 (3H, *d*, *J* = 2.5 Hz), 0.97–1.10 (1H, *m*), 1.27–1.48 (3H, *m*), 1.63–1.99 (1H, *m*), 2.62 (1H, *dt*, *J* = 3.5, 12.2 Hz), 3.65–3.71 (1H, *m*), 7.98 (3H, *br s*); δ_C (100 MHz, DMSO–d_6) 20.3, 20.4, 22.9, 28.3, 32.2, 32.3,

36.1, 44.0, 48.2, 175.0. Anal. Calcd. for $C_{10}H_{20}ClNO_2$ (221.72): C, 54.17; H, 9.09; N, 6.32; Found: C, 54.38; H, 9.25; N, 5.98.

(1*S*,2*S*,4*S*)-2-Amino-4-isopropylcyclohexanecarboxylic acid hydrochloride (10B)

According to *General procedure 5*, **10B** was obtained as white crystals (1.99 g, 90%); mp 247–250 °C; $[\alpha]_D^{20}$ = –23.0 (*c* 0.25 in MeOH); δ_H (400 MHz, DMSO–d$_6$) 0.82 (6H, *d*, *J* = 6.9 Hz), 0.84–0.92 (1H, *m*), 1.16–1.28 (1H, *m*), 1.36–1.58 (4H, *m*), 1.74–1.83 (1H, *m*), 2.07–2.18 (1H, *m*), 2.96–3.03 (1H, *m*), 3.10–3.19 (1H, *m*), 8.10 (3H, *br s*), 12.10 (1H, *br s*); δ_C (100 MHz, DMSO–d$_6$) 20.3, 20.4, 25.3, 27.8, 30.6, 32.8, 41.1, 42.7, 50.8, 174.8. Anal. Calcd. for $C_{10}H_{20}ClNO_2$ (221.72): C, 54.17; H, 9.09; N, 6.32; Found: C, 54.30; H, 9.31; N, 6.10.

(1*R*,2*S*,4*S*)-2-Amino-4-isopropylcyclohexanecarboxylic acid hydrochloride (10C)

According to *General procedure 5*, **10C** was obtained as white crystals (2.35 g, 94%); mp 220–223 °C; $[\alpha]_D^{20}$ = +38.0 (*c* 0.25 in MeOH); δ_H (400 MHz, DMSO–d$_6$) 0.84 (6H, *d*, *J* = 6.8 Hz), 0.94 (1H, *ddd*, *J* = 3.2, 12.9, 25.7 Hz), 1.04–1.24 (2H, *m*), 1.31 (1H, *ddd*, *J* = 3.4, 13.1, 26.0 Hz), 1.39–1.49 (1H, *m*), 1.36–1.58 (4H, *m*), 1.63–1.73 (1H, *m*), 1.98–2.12 (2H, *m*), 2.40 (1H, *ddd*, *J* = 3.9, 11.2, 14.9 Hz), 3.08–3.21 (1H, *m*), 8.10 (3H, *br s*), 12.80 (1H, *br s*); δ_C (100 MHz, DMSO–d$_6$) 20.3, 20.4, 28.4, 29.2, 32.7, 33.5, 42.0, 46.8, 51.2, 175.3. Anal. Calcd. for $C_{10}H_{20}ClNO_2$ (221.72): C, 54.17; H, 9.09; N, 6.32; Found: C, 54.35; H, 9.23; N, 5.99.

(1*R*,2*R*,4*S*)-2-Amino-4-isopropylcyclohexanecarboxylic acid hydrochloride (10D)

According to *General procedure 5*, **10D** was obtained as white crystals (2.03 g, 92%); mp 224–227 °C; $[\alpha]_D^{20}$ = –41.0 (*c* 0.125 in MeOH); δ_H (400 MHz, DMSO–d$_6$) 0.81 (3H, *d*, *J* = 6.0 Hz), 0.84 (3H, *d*, *J* = 6.0 Hz), 1.12–1.24 (1H, *m*), 1.38–1.52 (3H, *m*), 1.58–1.72 (2H, *m*), 1.75–1.90 (2H, *m*), 2.72 (1H, *dd*, *J* = 5.0, 9.9 Hz), 3.50–3.60 (1H, *m*), 8.25 (3H, *br s*), 12.75 (1H, *br s*); δ_C (100 MHz, DMSO–d$_6$) 20.6, 20.7, 23.0, 26.2, 30.3, 30.7, 37.4, 43.5, 47.8, 174.4. Anal. Calcd. for $C_{10}H_{20}ClNO_2$ (221.72): C, 54.17; H, 9.09; N, 6.32; Found: C, 54.40; H, 9.21; N, 6.01.

X-ray crystallographic study of 10D: Crystallographic data were collected at 123 K with a Nonius-Kappa CCD area detector diffractometer, using graphite-monochromatized Mo-K_{α} radiation (λ = 0.71073 Å) as reported earlier [5]. The structure was solved by direct methods by use of the SHELXS-97 program [6] and full-matrix, least-squares refinements on F^2 were performed by use of the SHELXL-97 program [6]. The CH hydrogen atoms were included at fixed distances from their host atoms with the fixed displacement parameters. The NH and OH hydrogen atoms were refined isotropically and the hydrogen bonds formed by them control the crystal packing of **10D**. The ORTEP plot was drawn with *ORTEP-3 for Windows* [7]. The deposition number CCDC 1011603 contains the supplementary crystallographic data for this paper. These data can be obtained free of charge from The Cambridge Crystallographic Data Centre via http://www.ccdc.cam.ac.uk/conts/retrieving.html (or from the Cambridge Crystallographic Data Centre, 12 Union Road, Cambridge CB2 1EZ, UK; Fax: (internat.) + 44-1223-336-033; E-mail: deposit@ccdc.cam.ac.uk)

Crystal data for 10D, $C_{10}H_{20}ClNO_2$, M_r = 221.72.50, orthorhombic, space group $P2_12_12_1$ (no. 9), a = 6.6955(1), b = 7.9887(2), c = 22.8357(3) Å, α = β = δ = 90°, V = 1221.44(4) Å^3, T = 123 K, Z = 4, μ(Mo-K_{α}) = 0.291 mm^{-1}. Total 3023 refelections, unique 2398. Refinement of 2315 reflections (145 parameters) with $I > 2\delta(I)$. converged at final $R1$ = 0.0267 ($R1$ all data = 0.0283), $wR2$ = 0.0629 ($wR2$ all data = 0.0640), GOF = 1.074, Flack parameter = 0.00(3).

References

1. Kitahara, T.; Horiguchi, A.; Mori, K. *Tetrahedron* **1988**, *44*, 4713–4720.

2. Cooke, R. G.; Macbeth, A. K.; Swanson, T. B. *J. Chem. Soc.* **1940**, 808–810.

3. Suga, T.; Sugimoto, M.; Fujita, K.; Matsuura, T. *Bull. Chem. Soc. Jpn.* **1966**, *39*, 2546–2547.

4. Mori, K. *Tetrahedron: Asymmetry* **2006**, *17*, 2133–2142.

5. Kanizsai, I.; Szakonyi, Z.; Sillanpää, R.; D'Hooghe, M.; De Kimpe, N.; Fülöp, F. *Tetrahedron – Asymmetry* **2006**, *17*, 2857–2863.

6. Sheldrick, G. M. *A short history of SHELX. Acta Cryst.* **2008**, *A64*, 112–122.

7. Farrugia, L. J. *J. Appl. Cryst.* **1997**, ***30***, 565.

^{1}H, ^{13}C NMR and HMBC of **3** ($CDCl_3$)

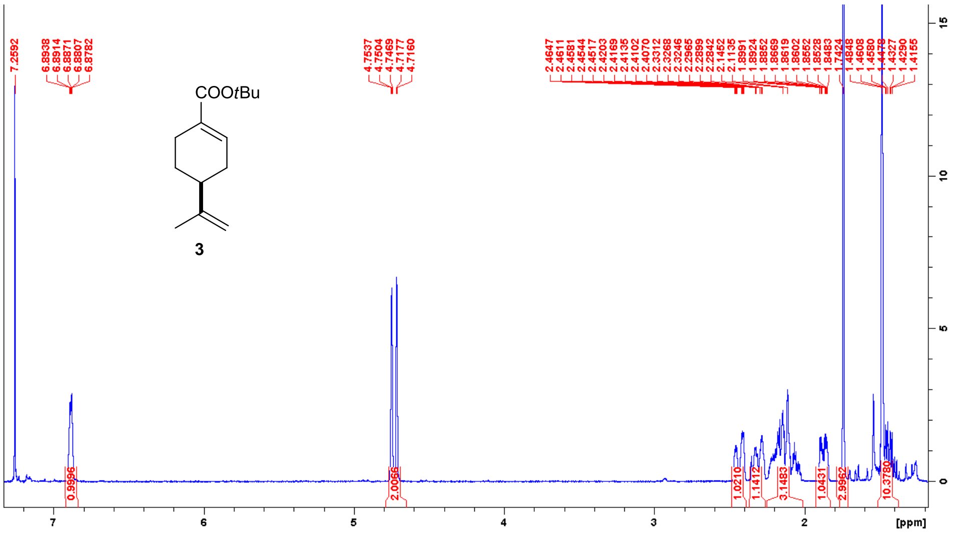

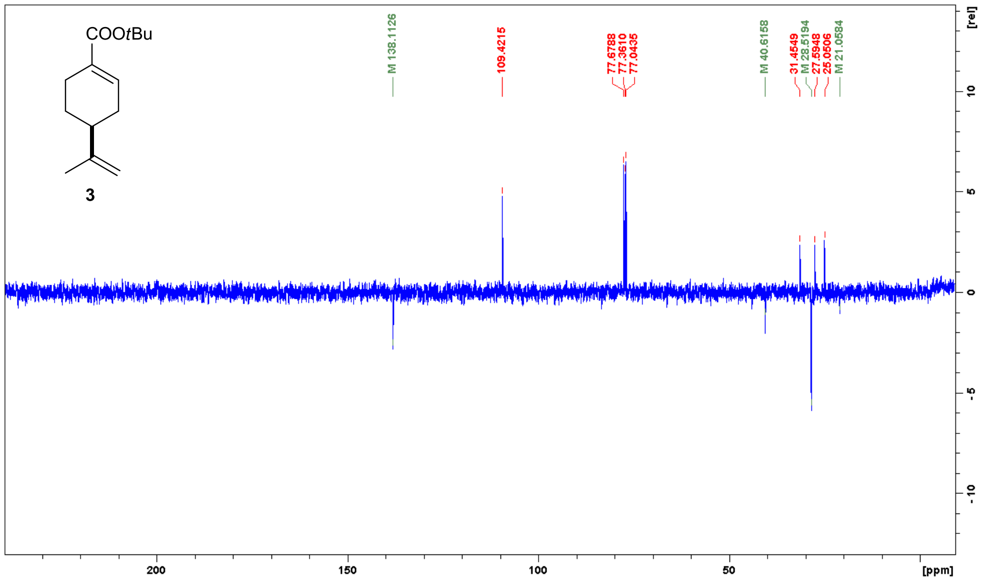

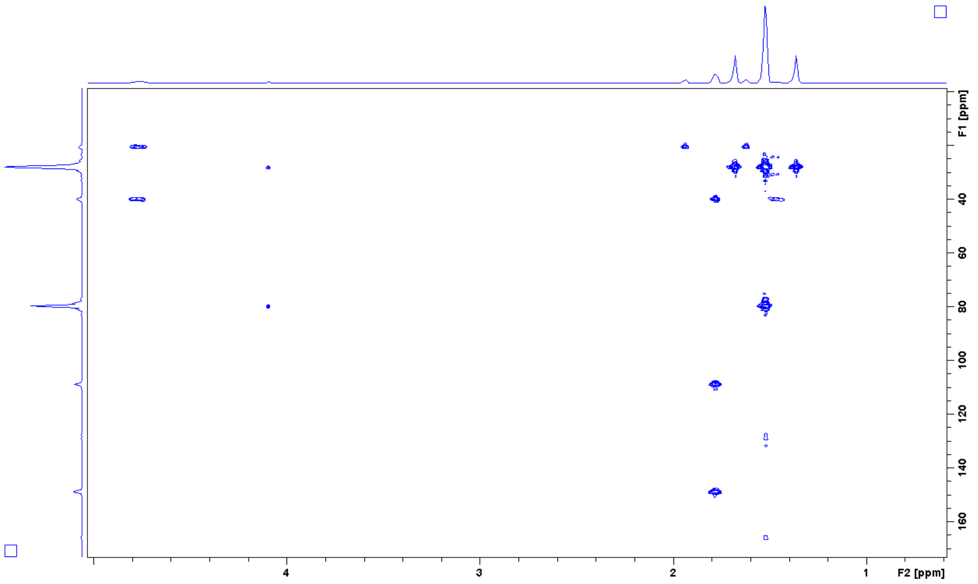

^{1}H and ^{13}C NMR of **6** ($CDCl_3$)

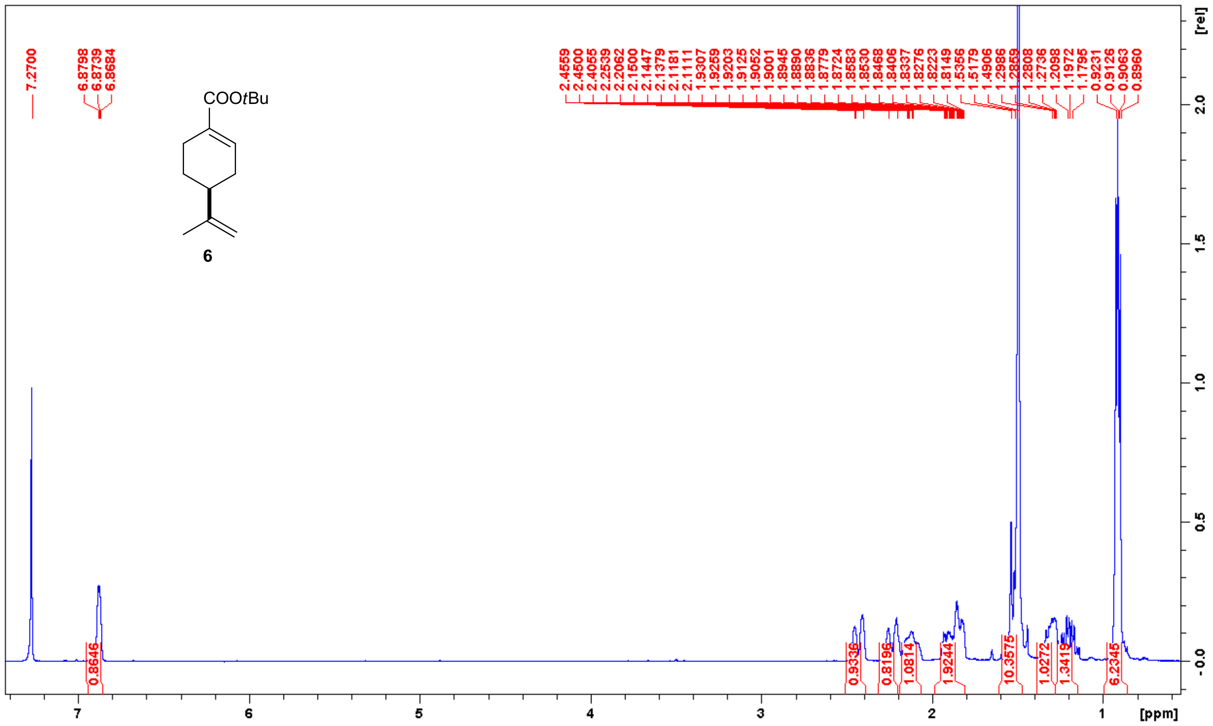

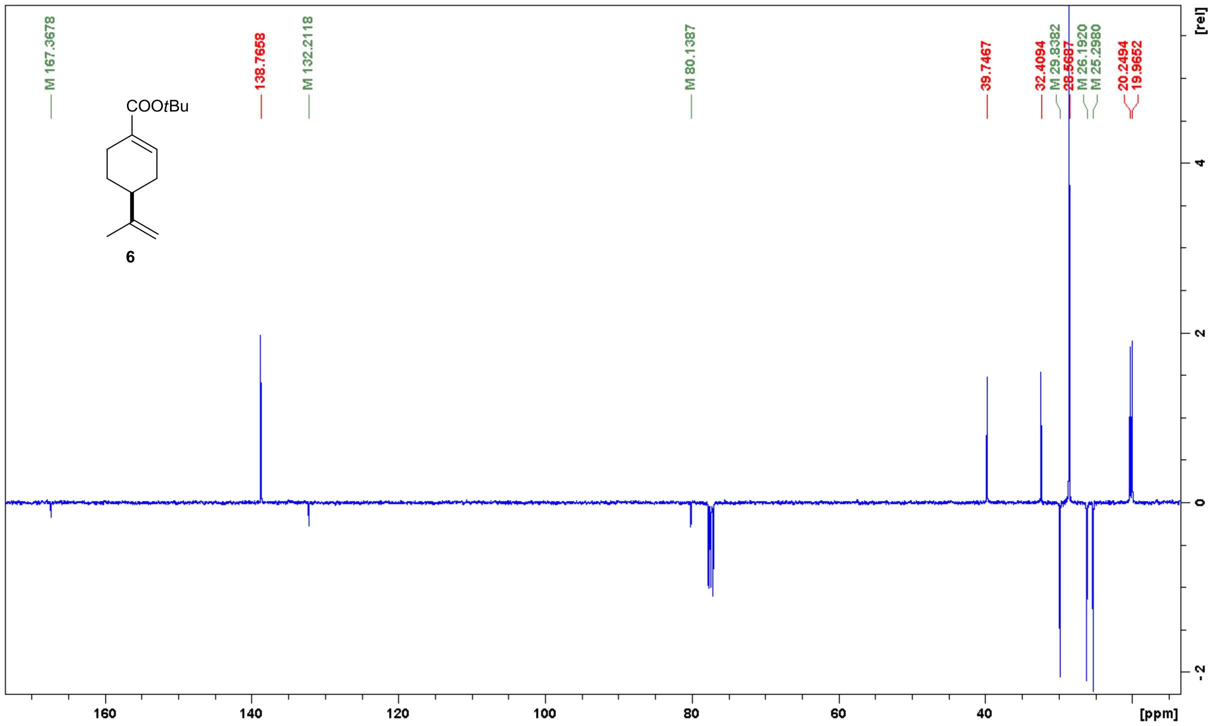

Informative part of the ^{1}H NMR of the crude product **7A-D** ($CDCl_3$)

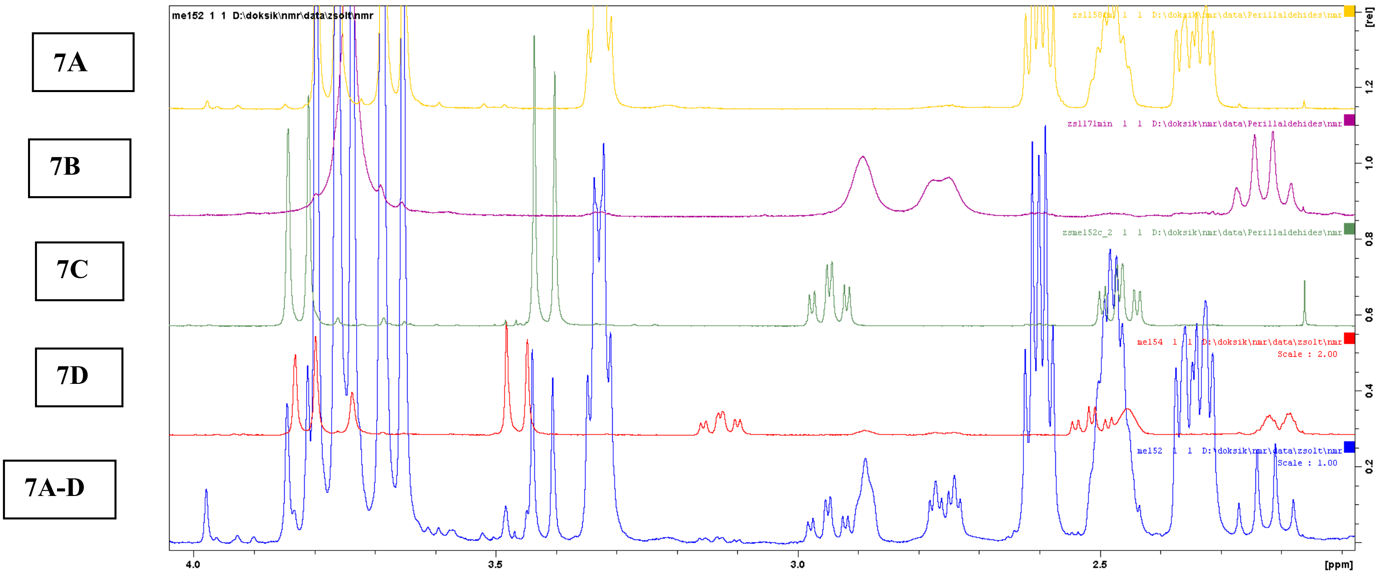

^{1}H and ^{13}C NMR of **7A** ($CDCl_3$)

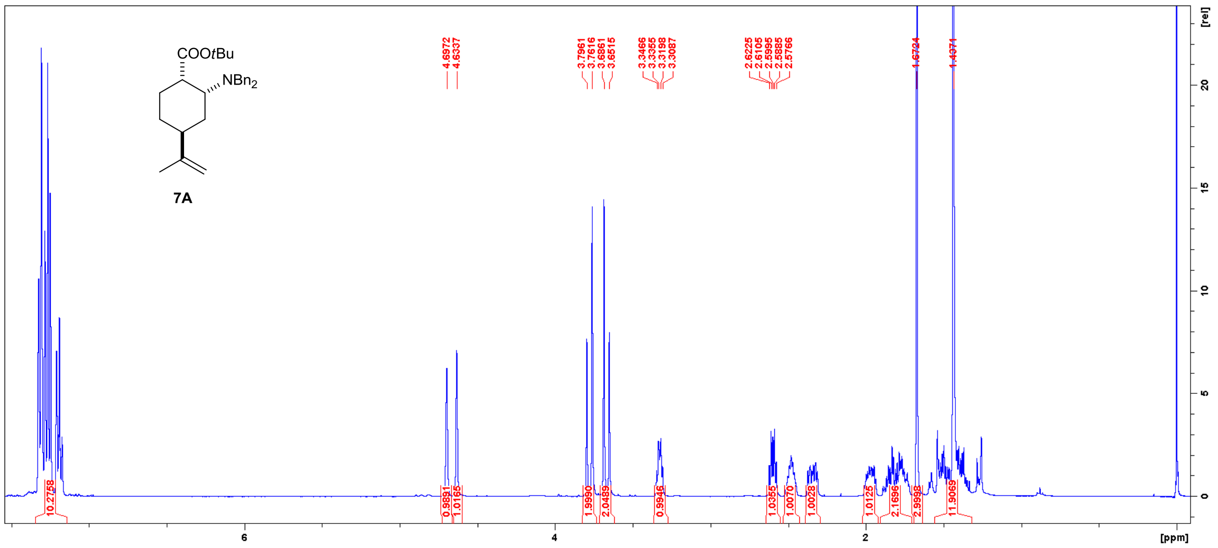

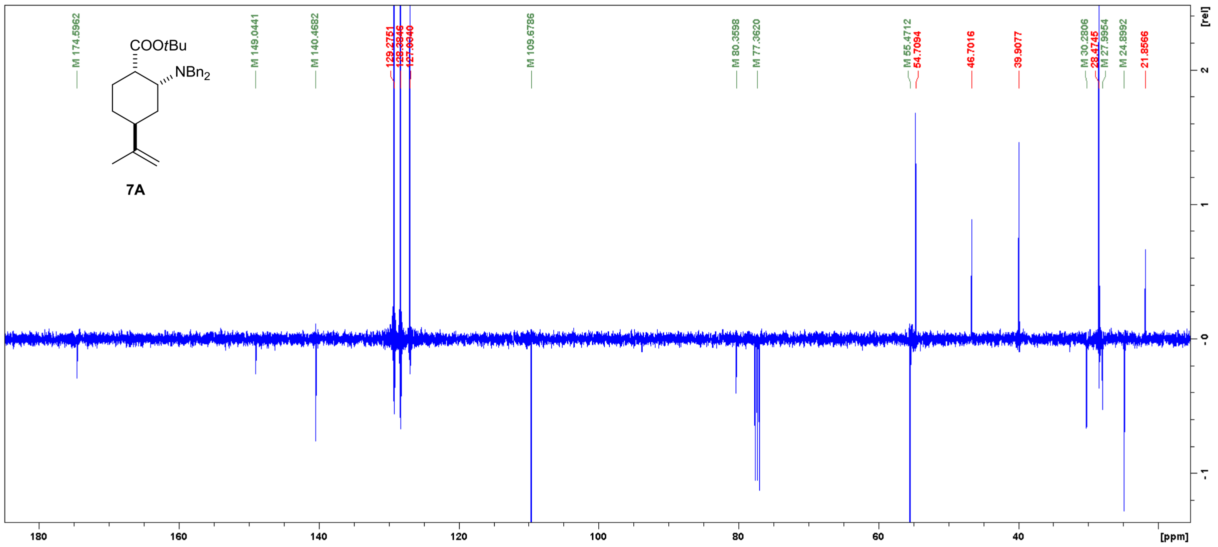

^{1}H, ^{13}C and HMBC NMR of **7B** ($CDCl_3$)

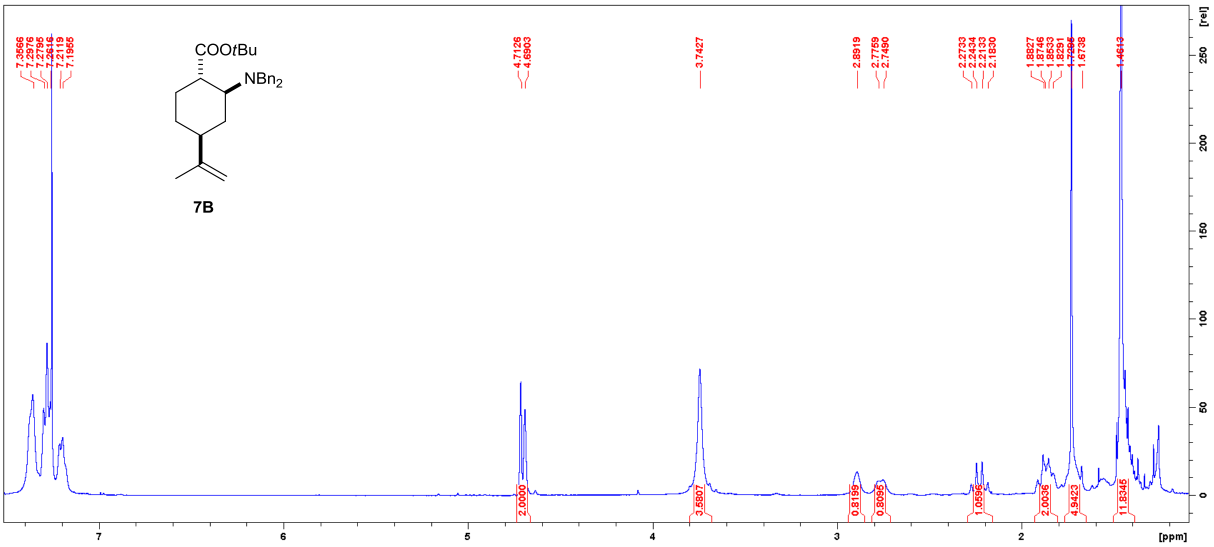

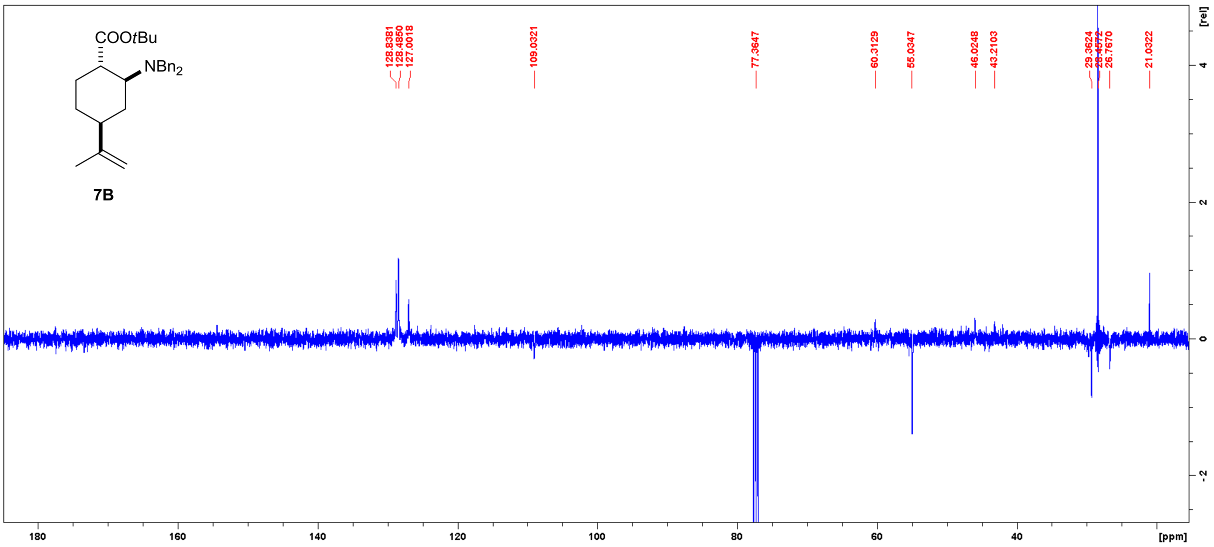

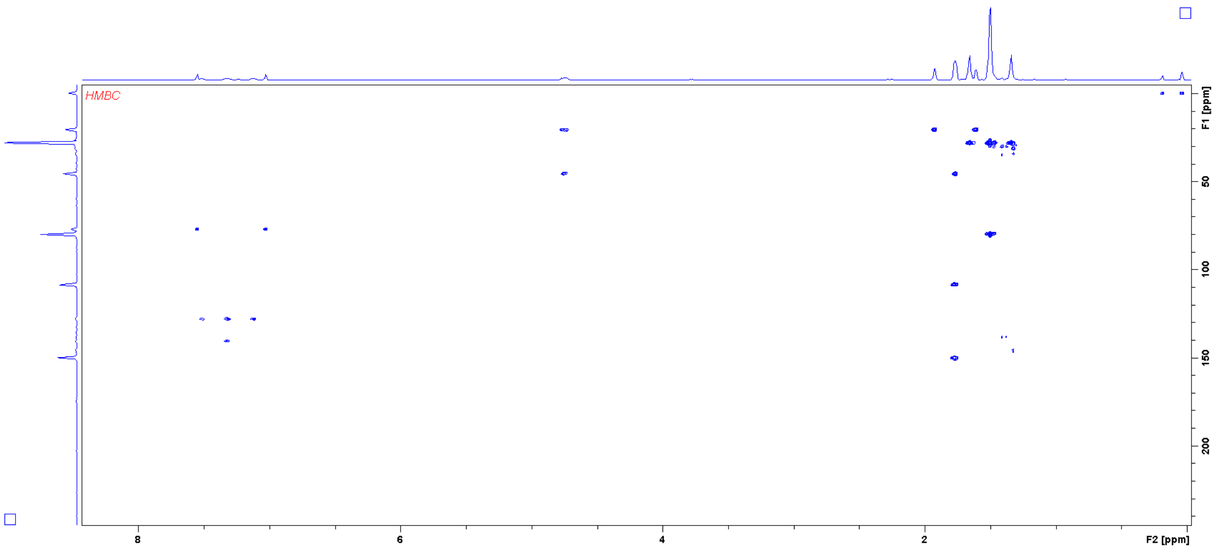

^{1}H and ^{13}C NMR of **7C** ($CDCl_3$)

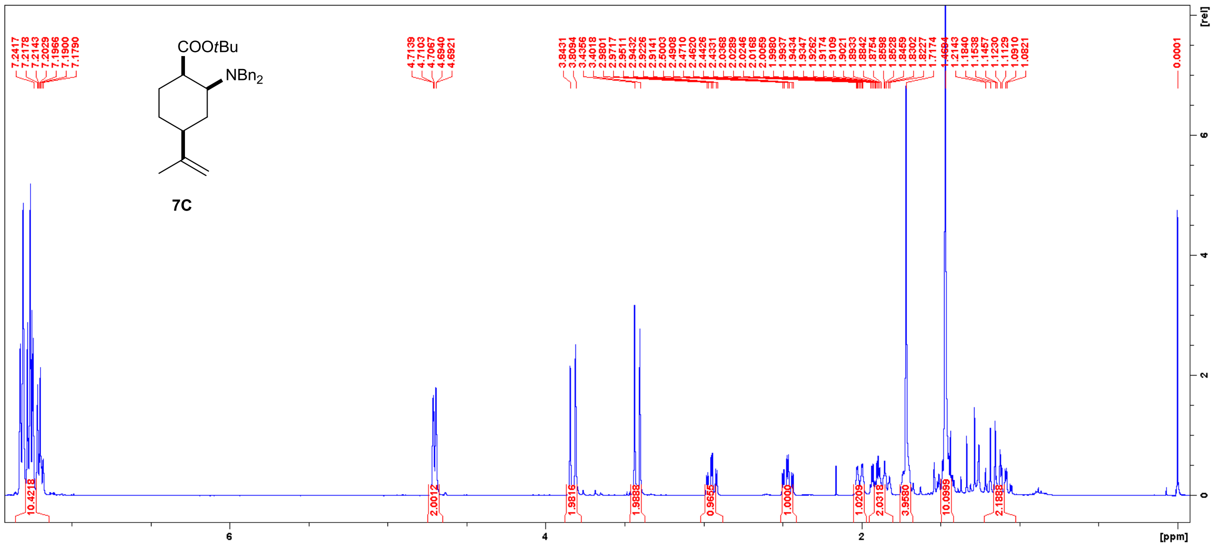

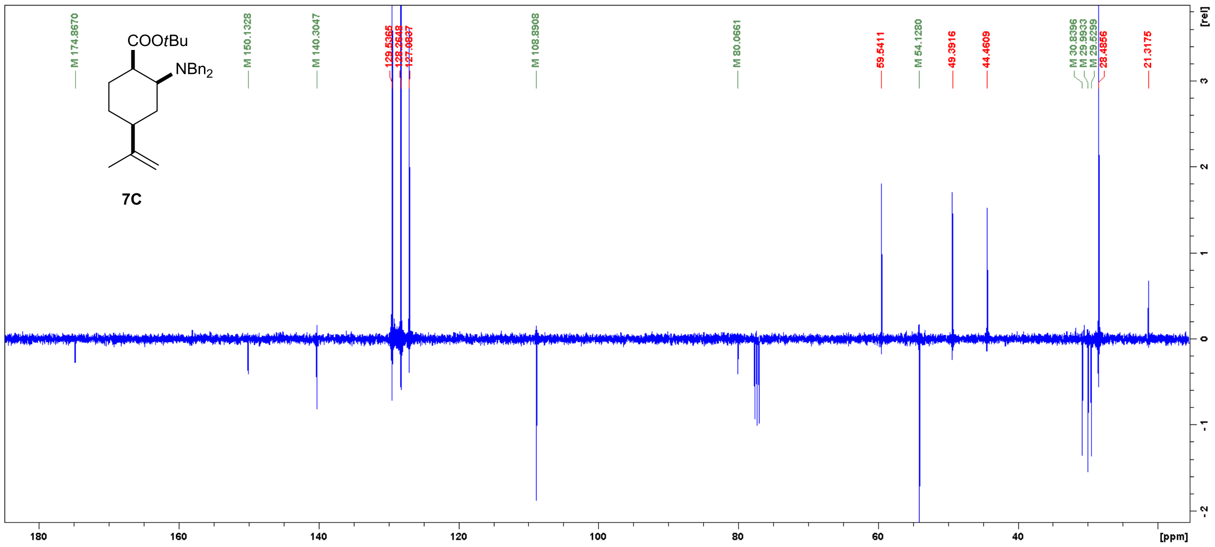

^{1}H and ^{13}C NMR of **7D** ($CDCl_3$)

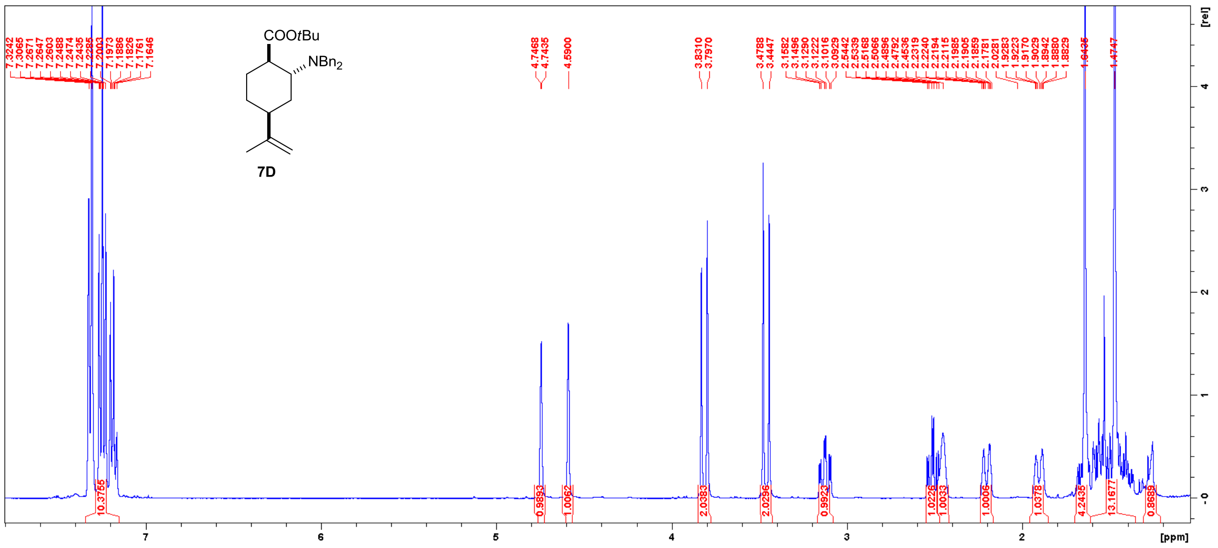

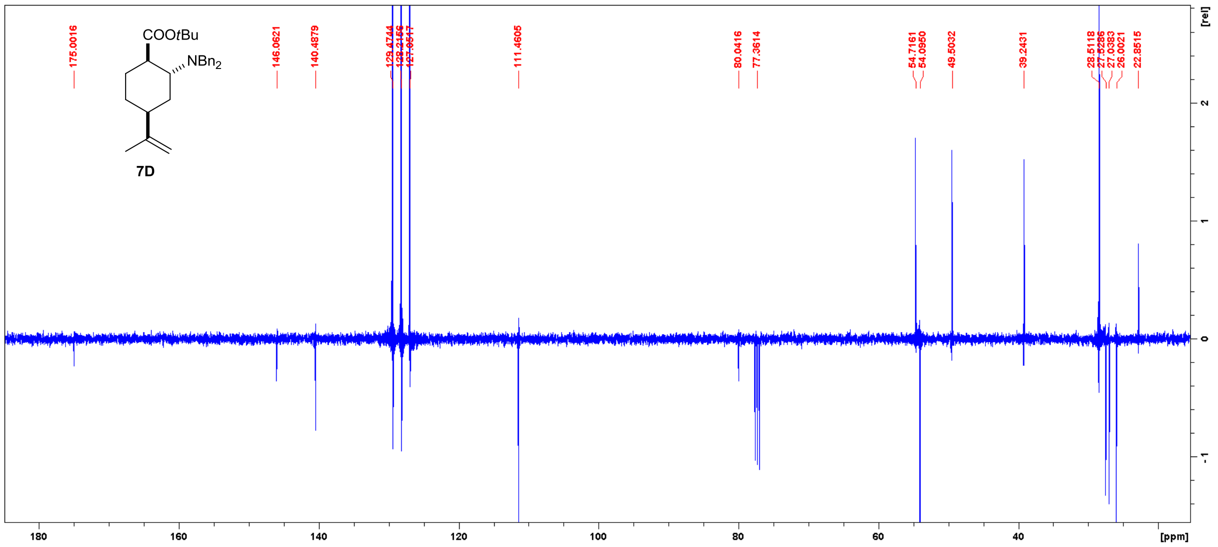

^{1}H, ^{13}C and HMBC NMR of **8A** ($CDCl_3$)

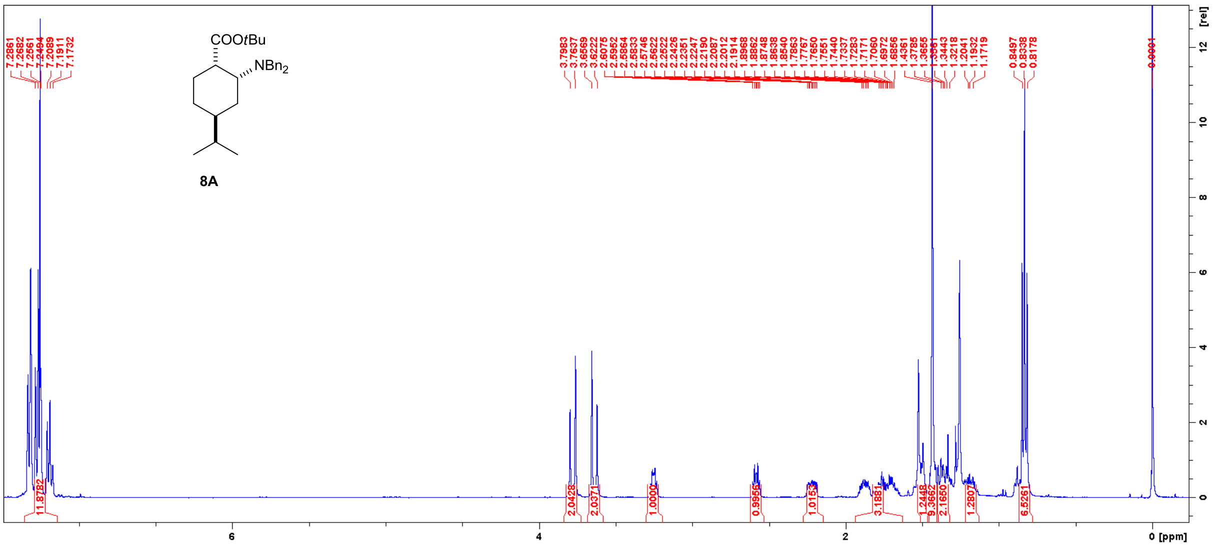

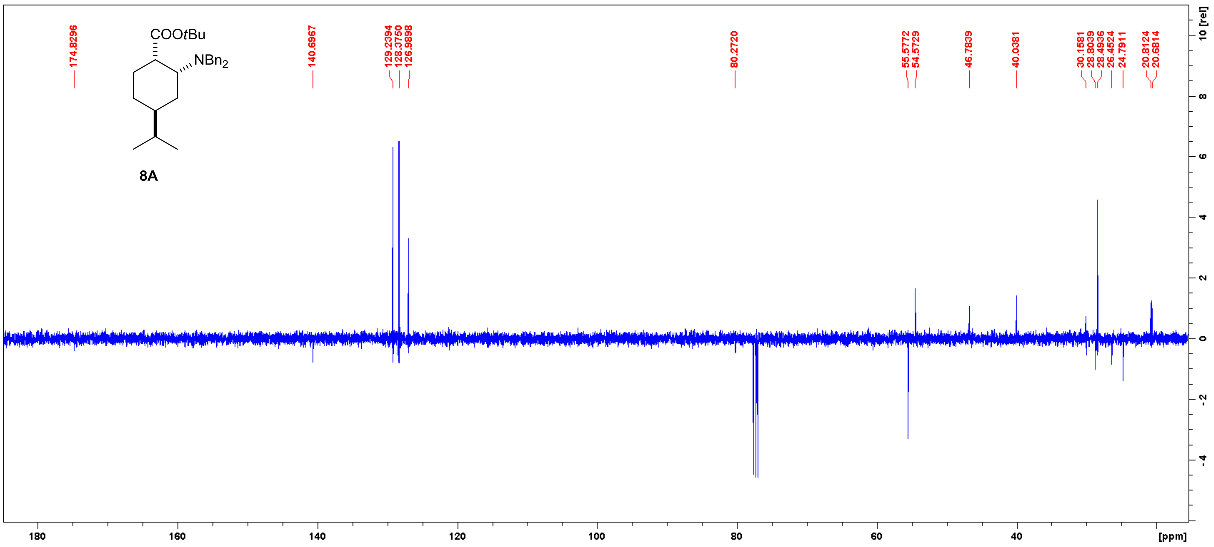

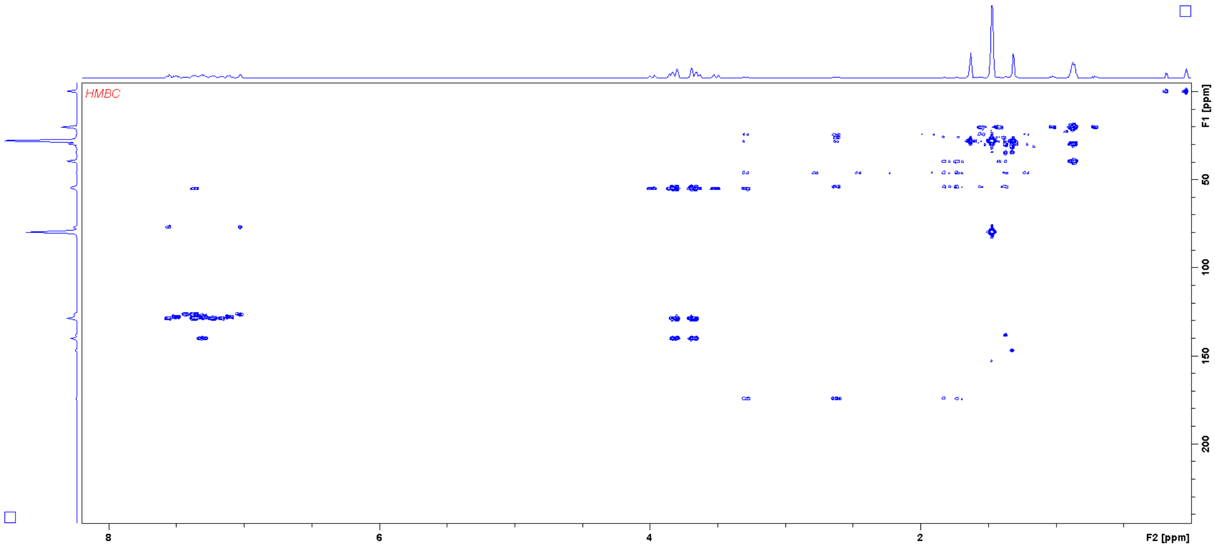

^{1}H and ^{13}C NMR of **8B** ($CDCl_3$)

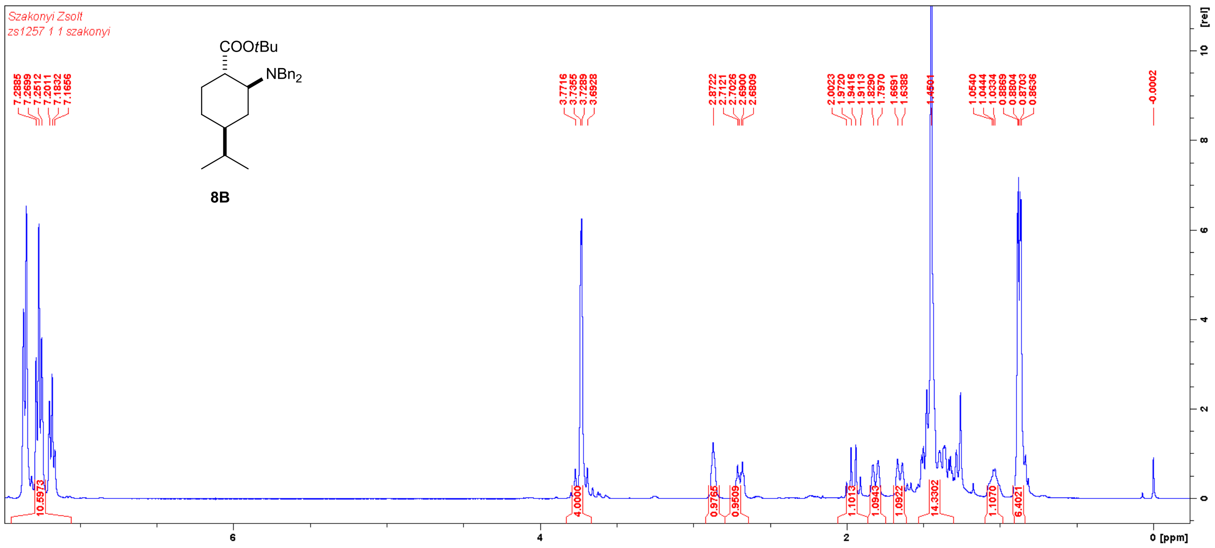

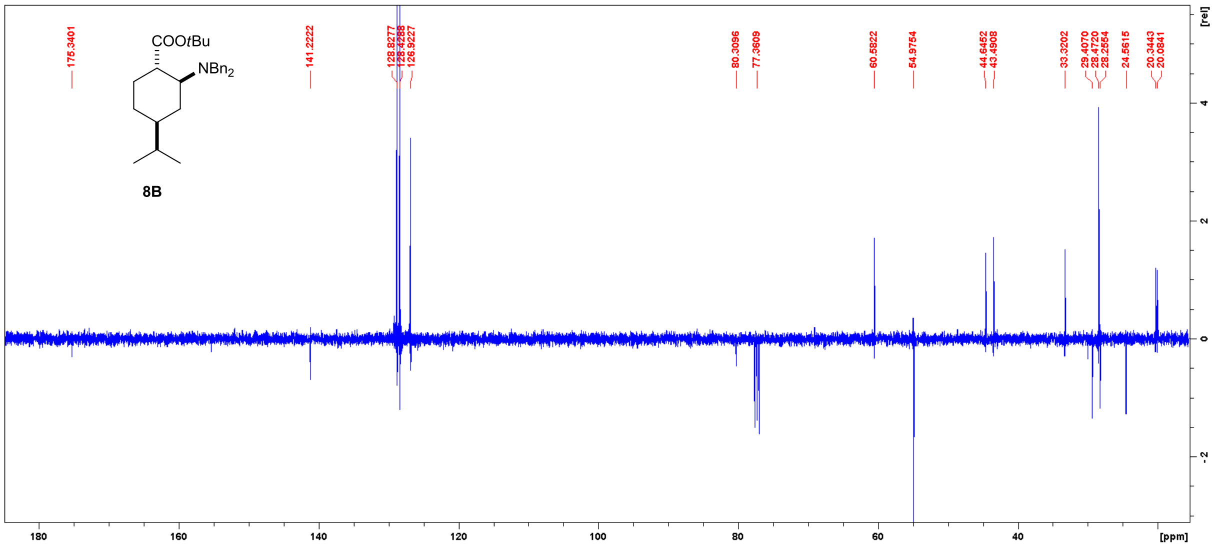

^{1}H and ^{13}C NMR of **8C** ($CDCl_3$)

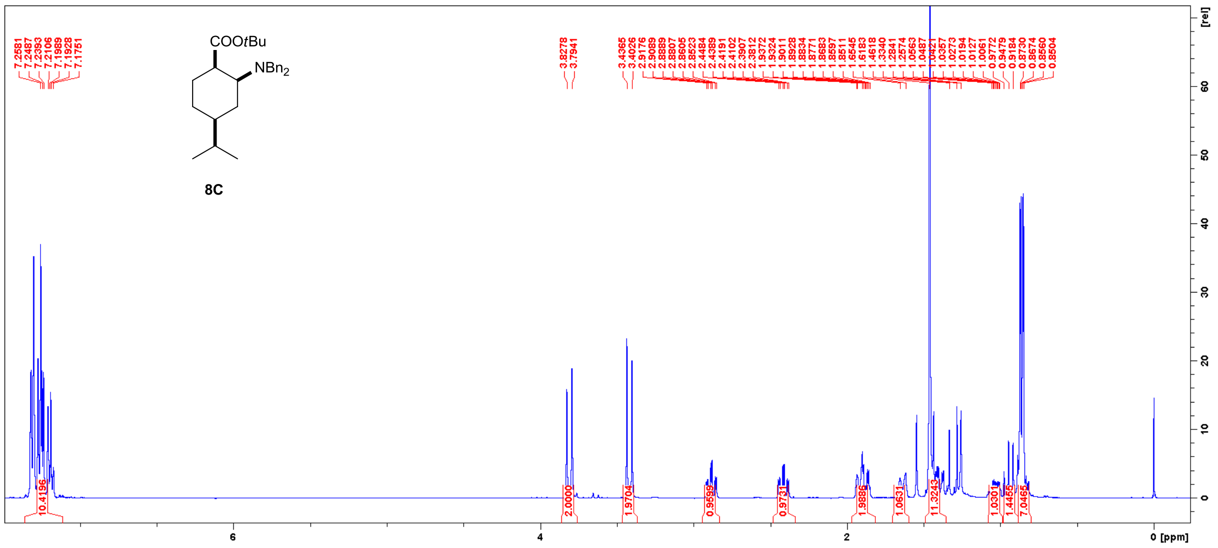

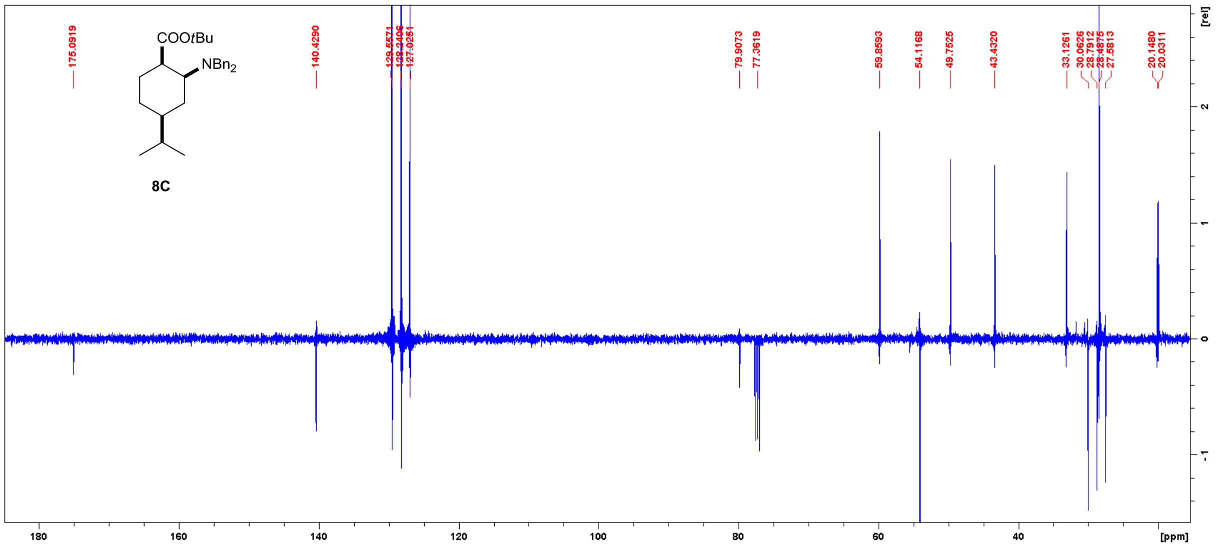

^{1}H, ^{13}C and HMBC NMR of **8D** ($CDCl_3$)

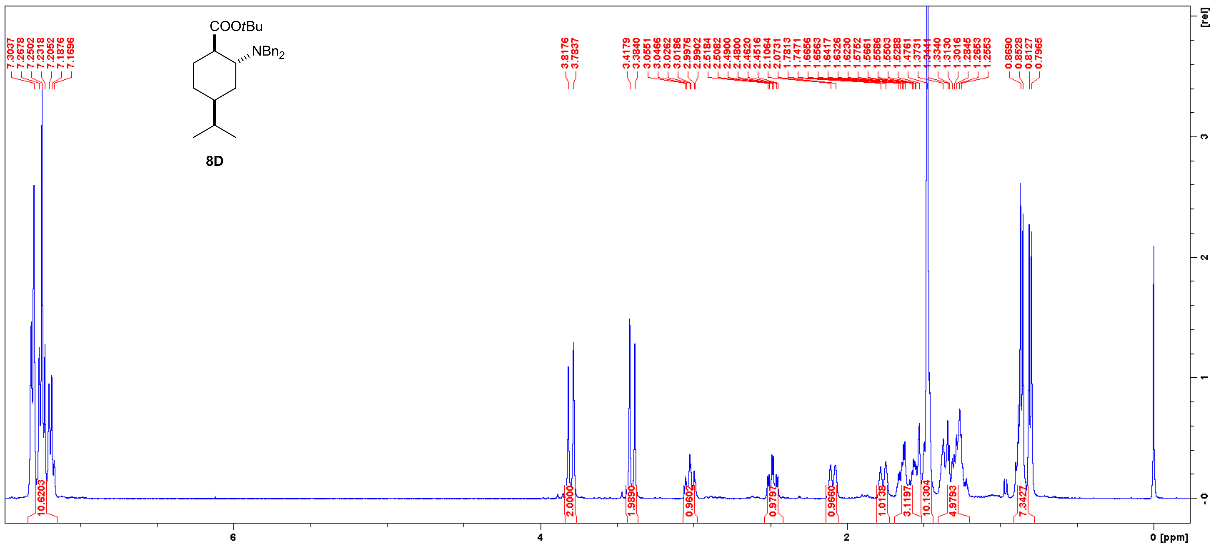

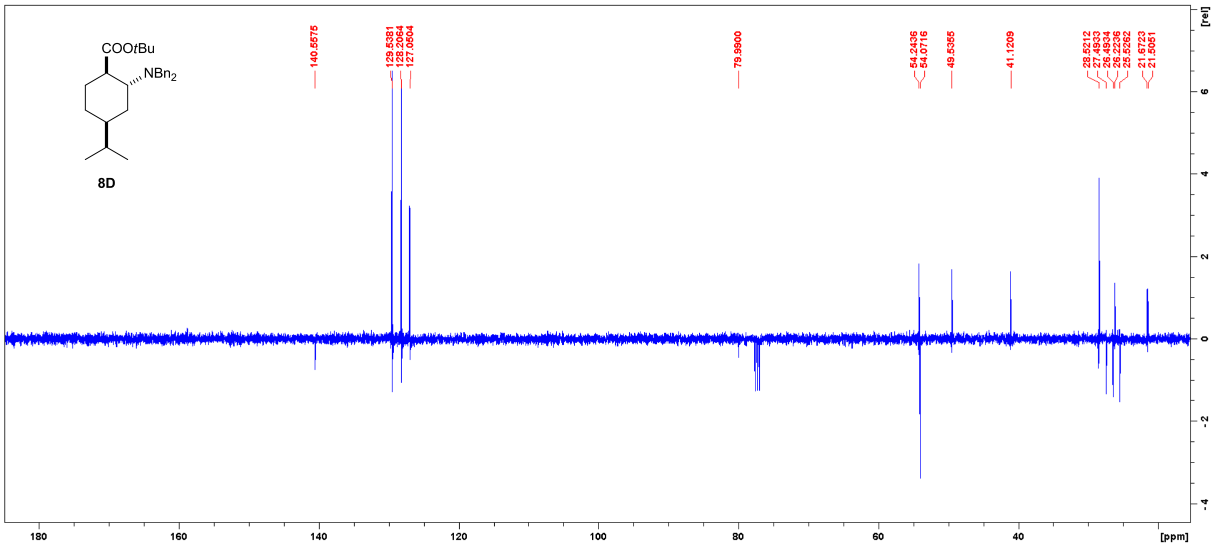

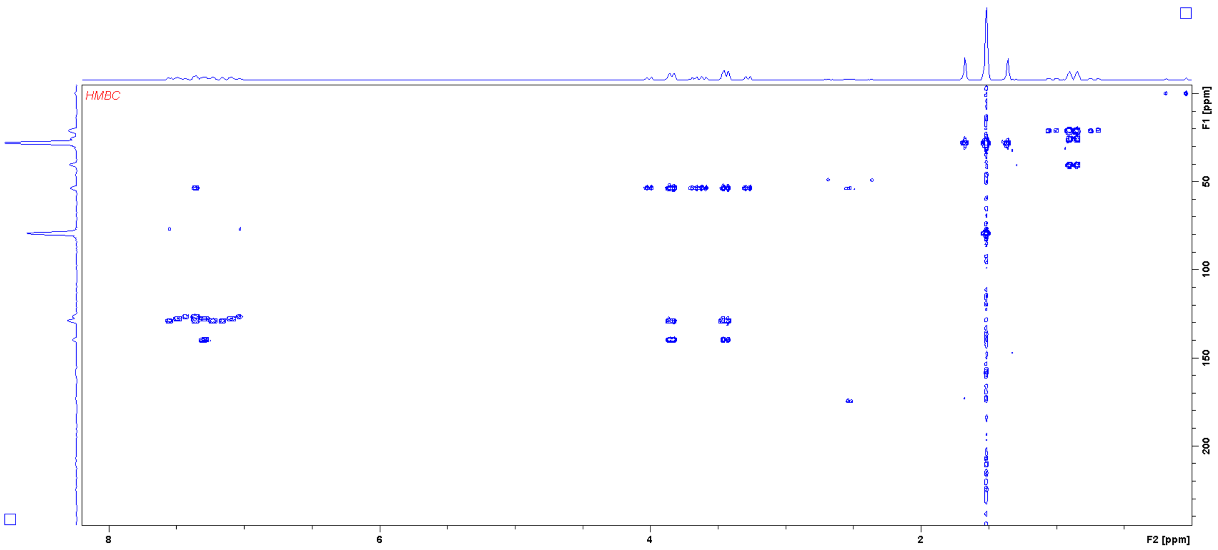

^{1}H and ^{13}C NMR of **9A** ($CDCl_3$)

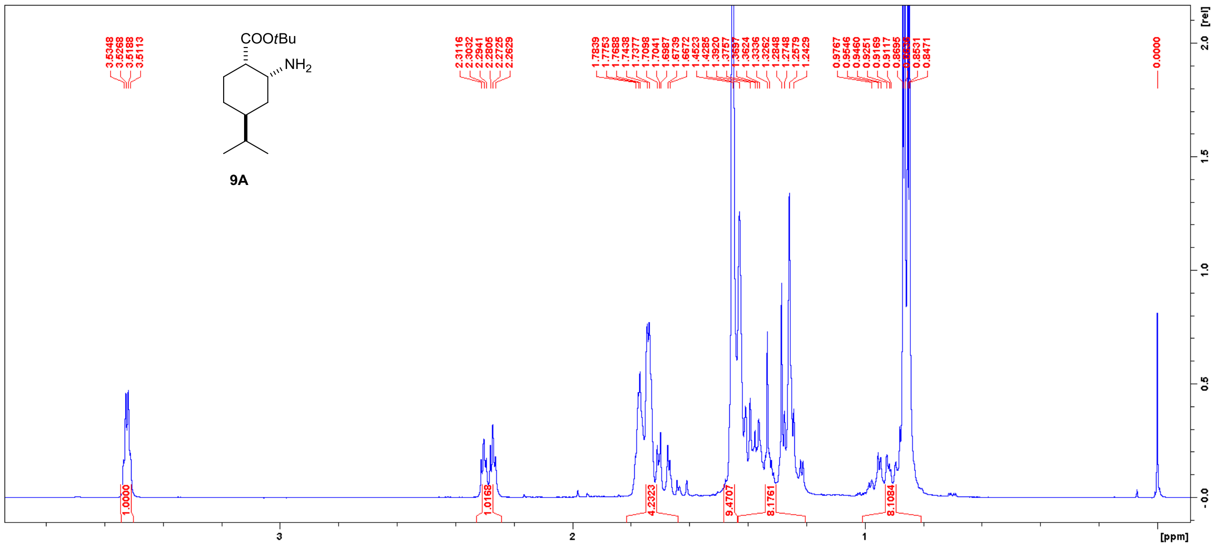

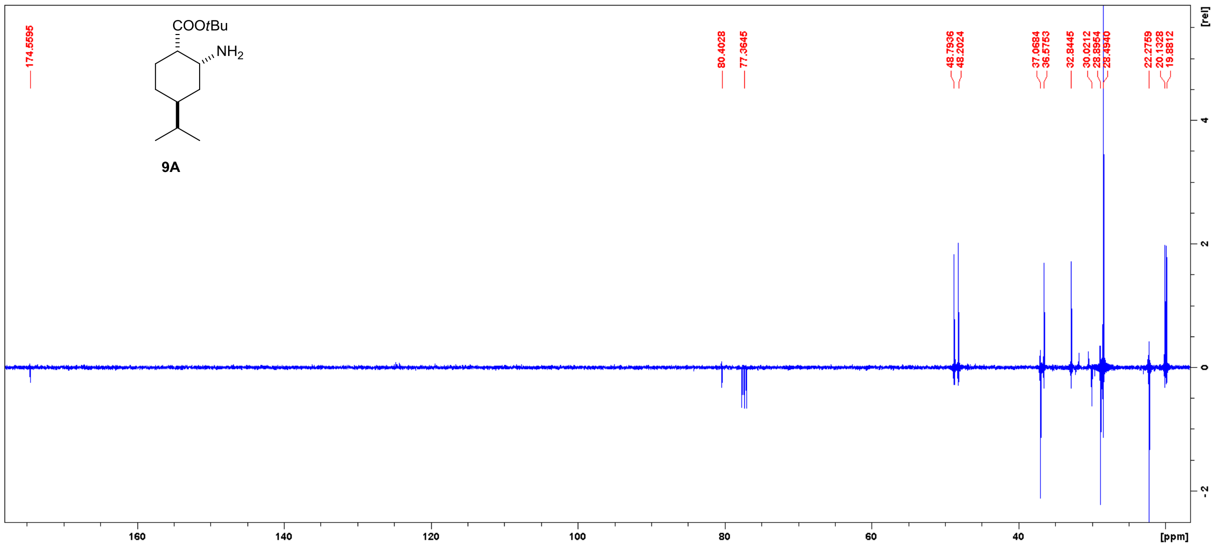

^{1}H and ^{13}C NMR of **9B** ($CDCl_3$)

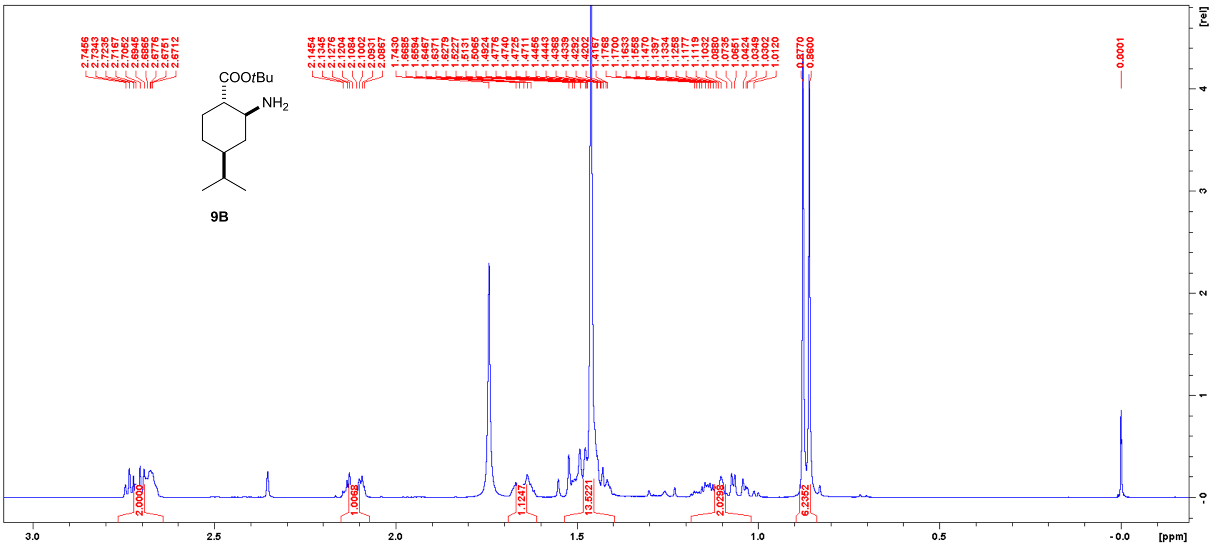

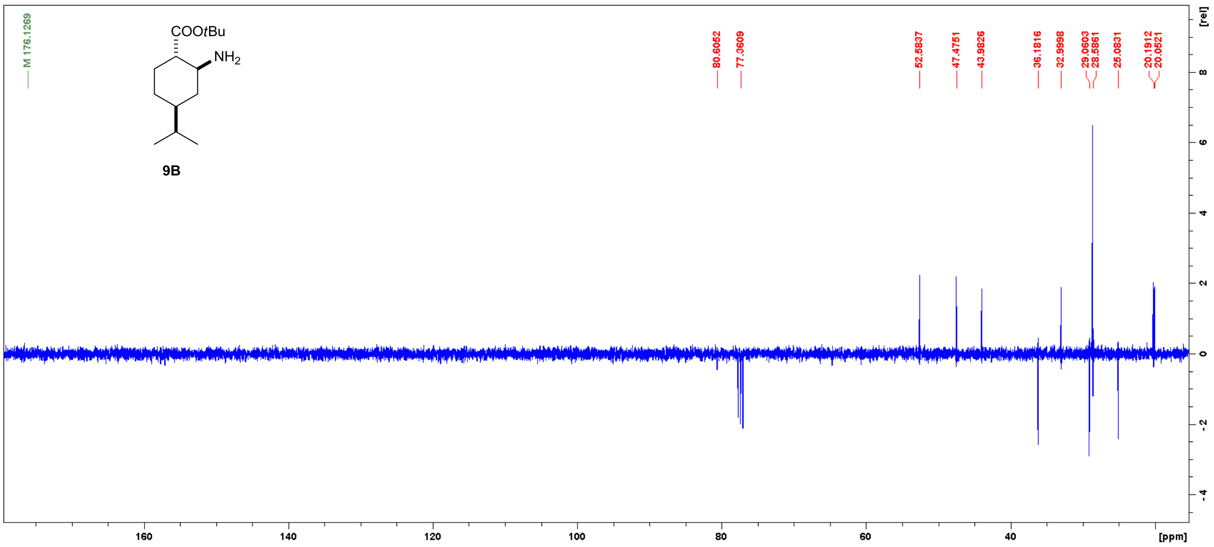

^{1}H and ^{13}C NMR of **9C** ($CDCl_3$)

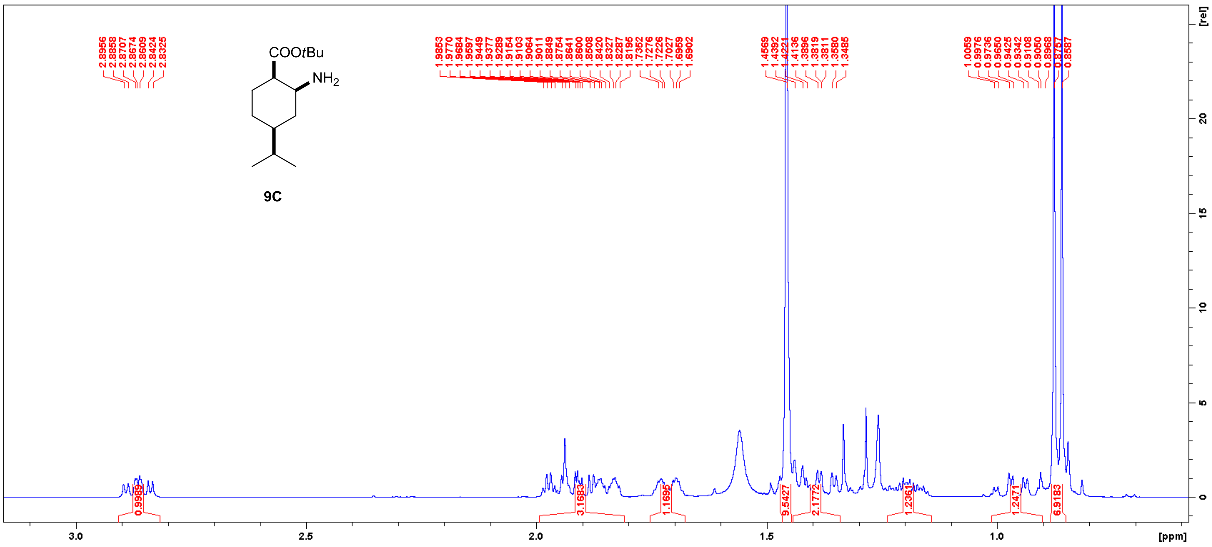

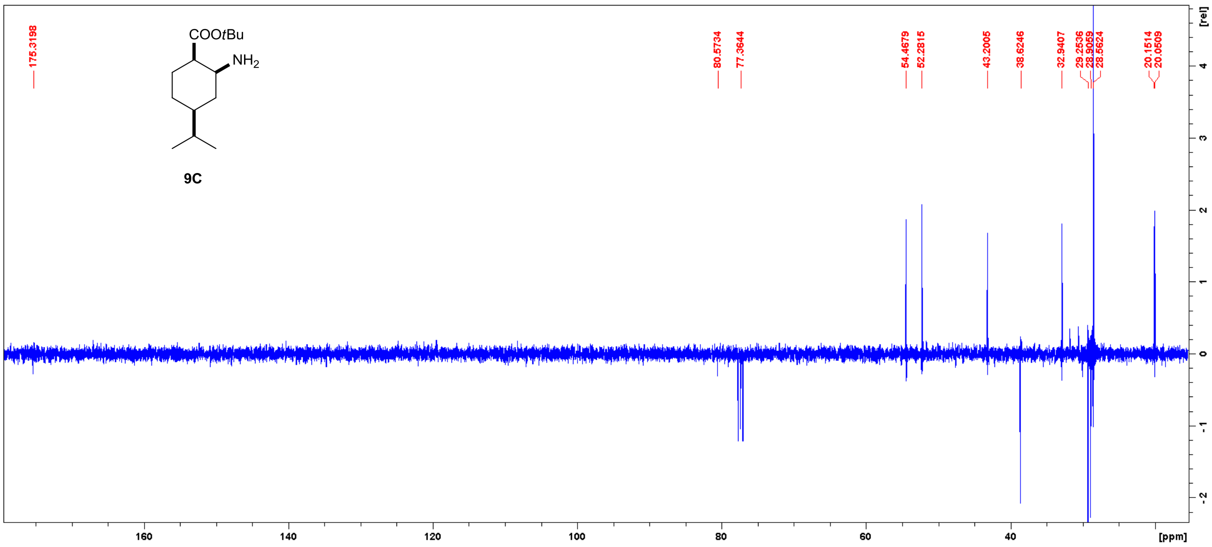

^{1}H and ^{13}C NMR of **9D** ($CDCl_3$)

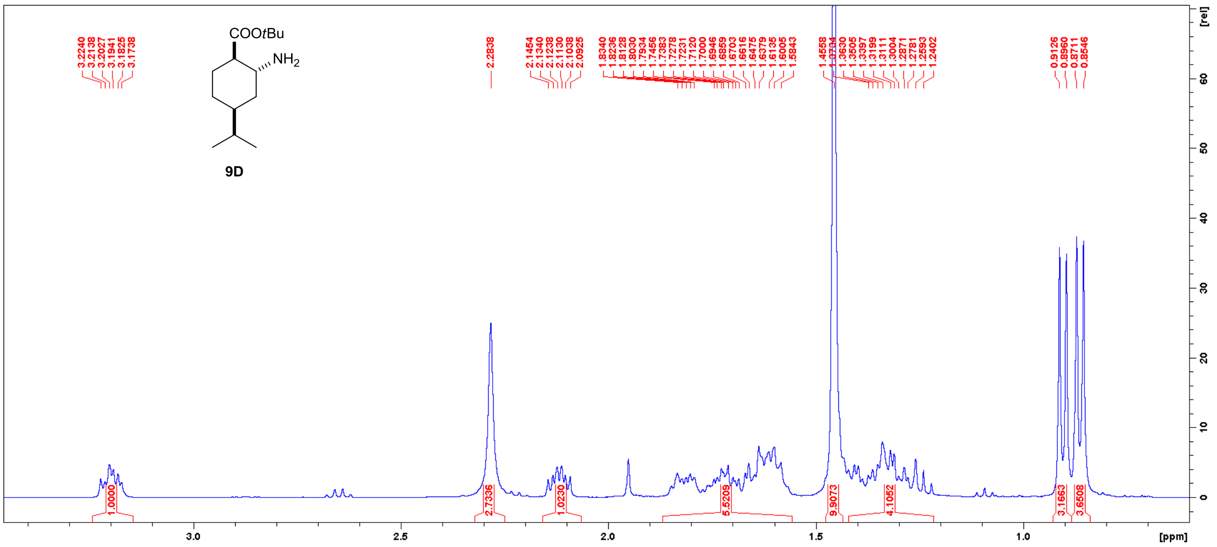

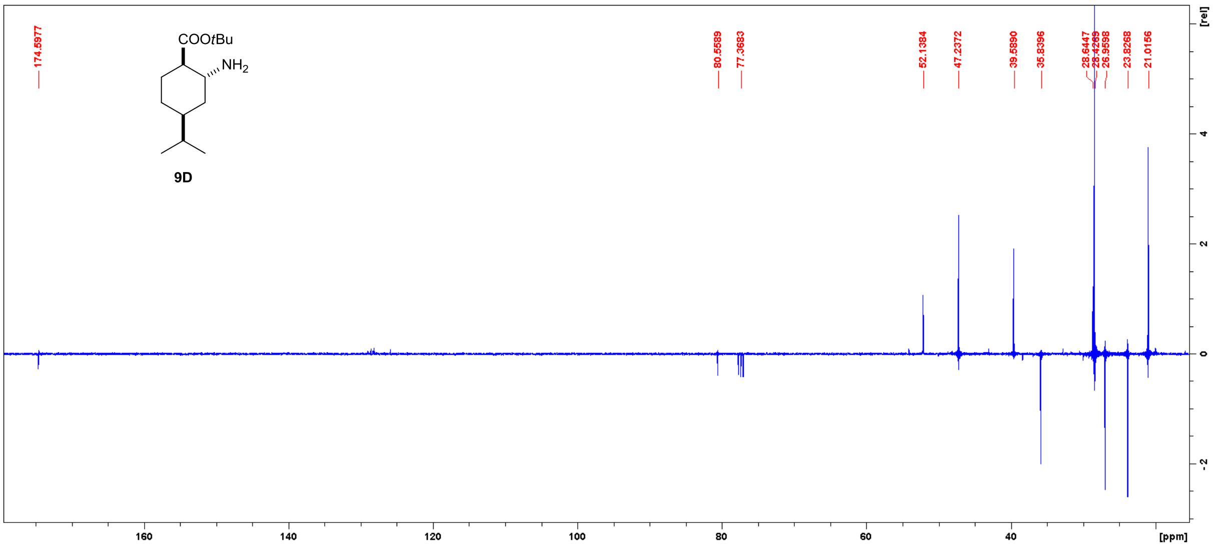

^{1}H and ^{13}C NMR of **10A** (DMSO–d_6)

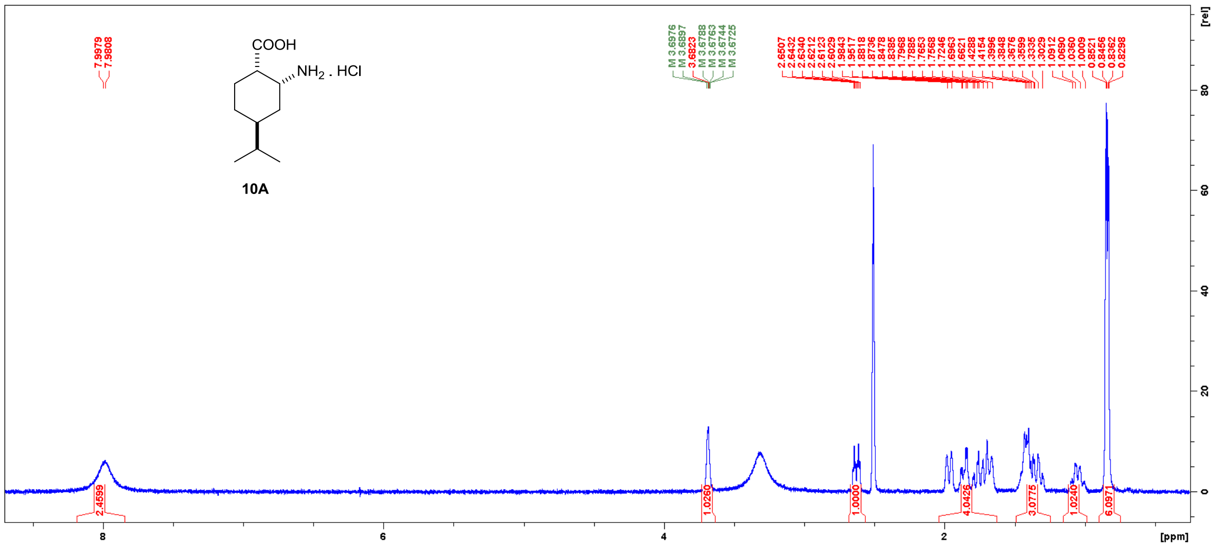

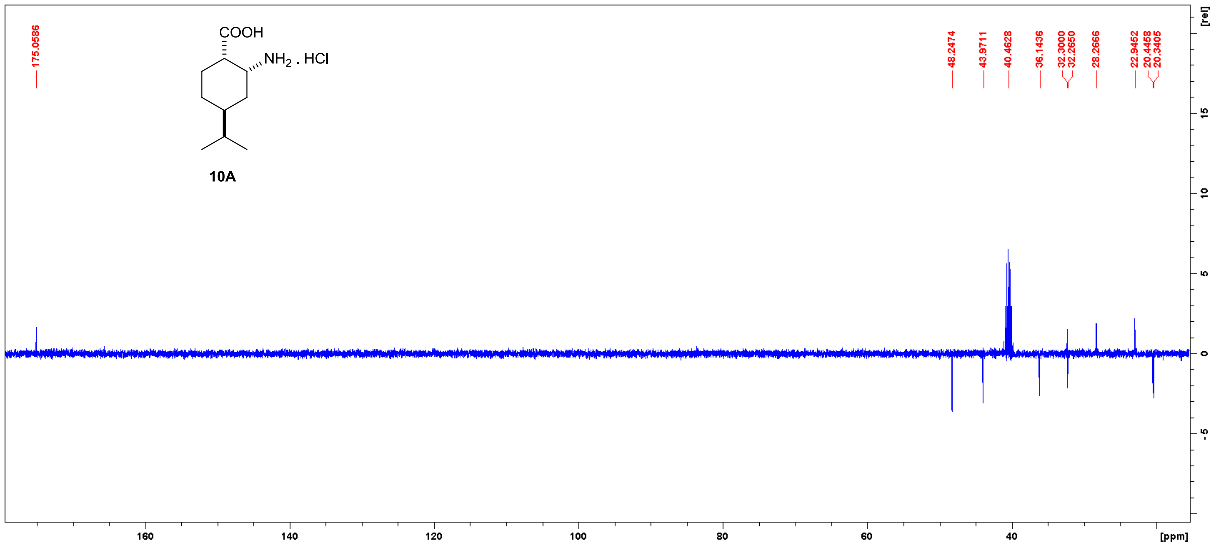

^{1}H and ^{13}C NMR of **10B** (DMSO–d_6)

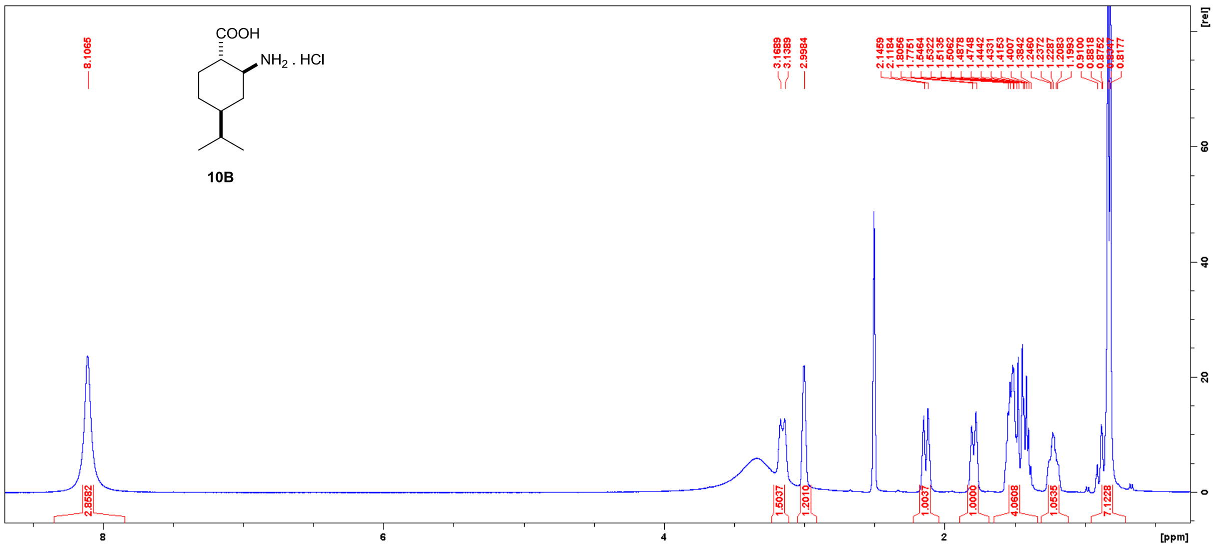

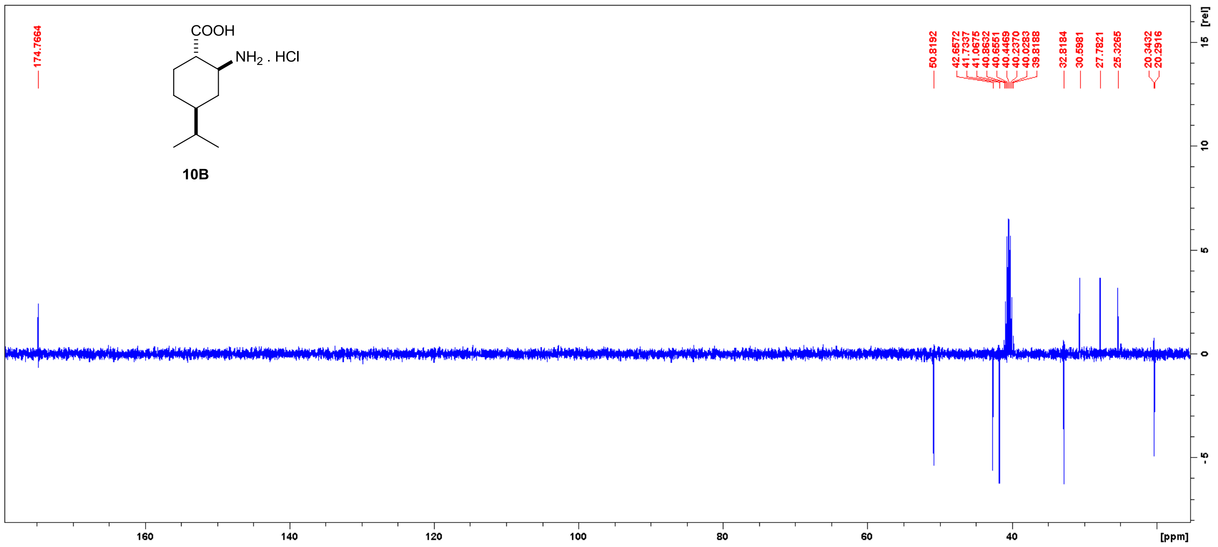

^{1}H and ^{13}C NMR of **10C** (DMSO–d_6)

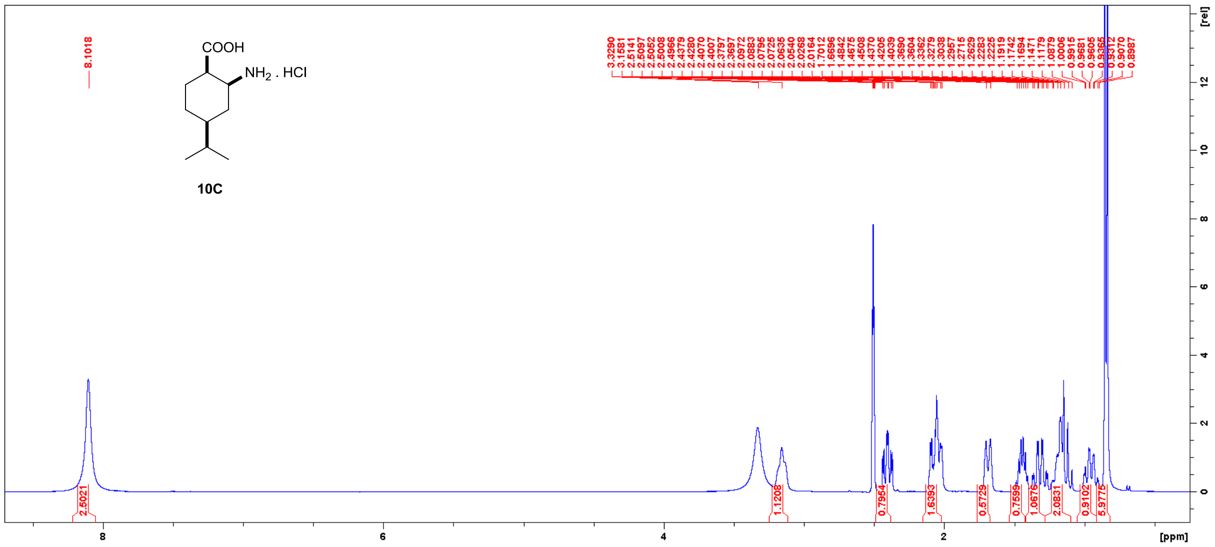

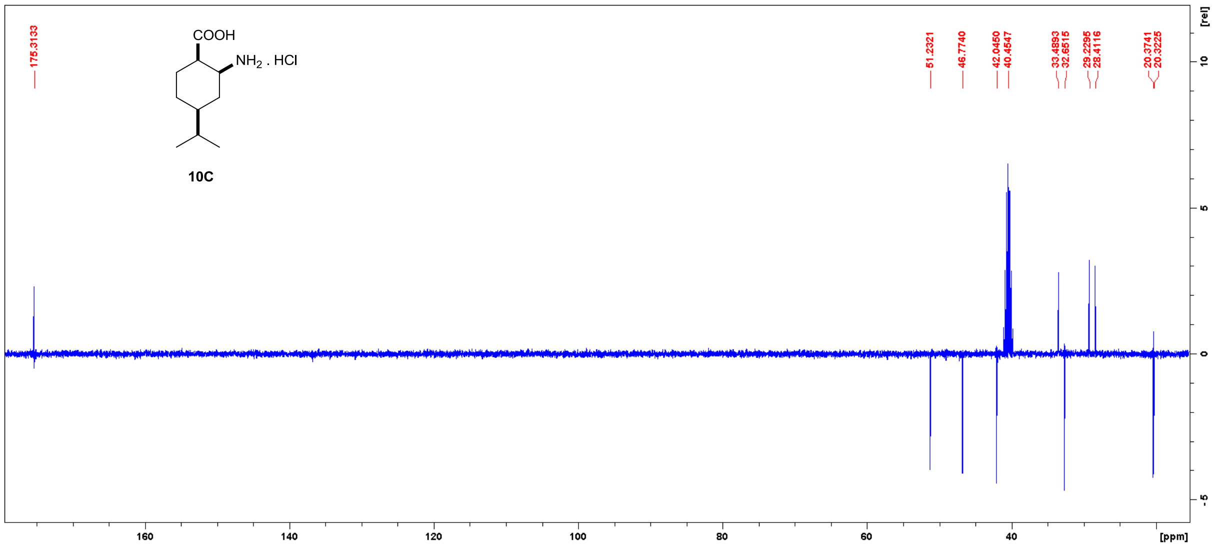

^{1}H and ^{13}C NMR of **10D** (DMSO–d_6)

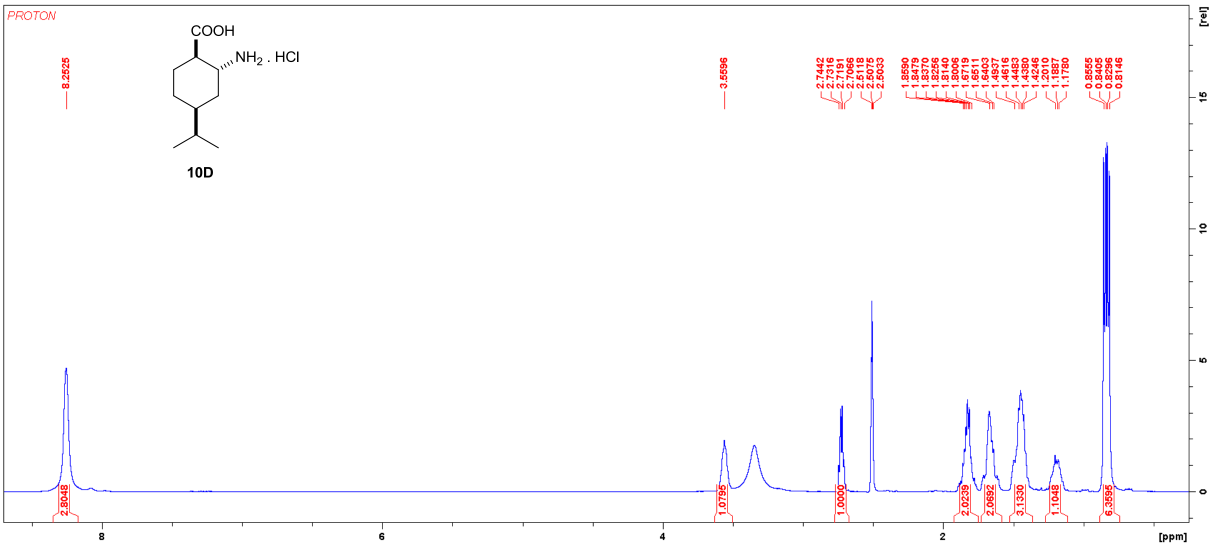

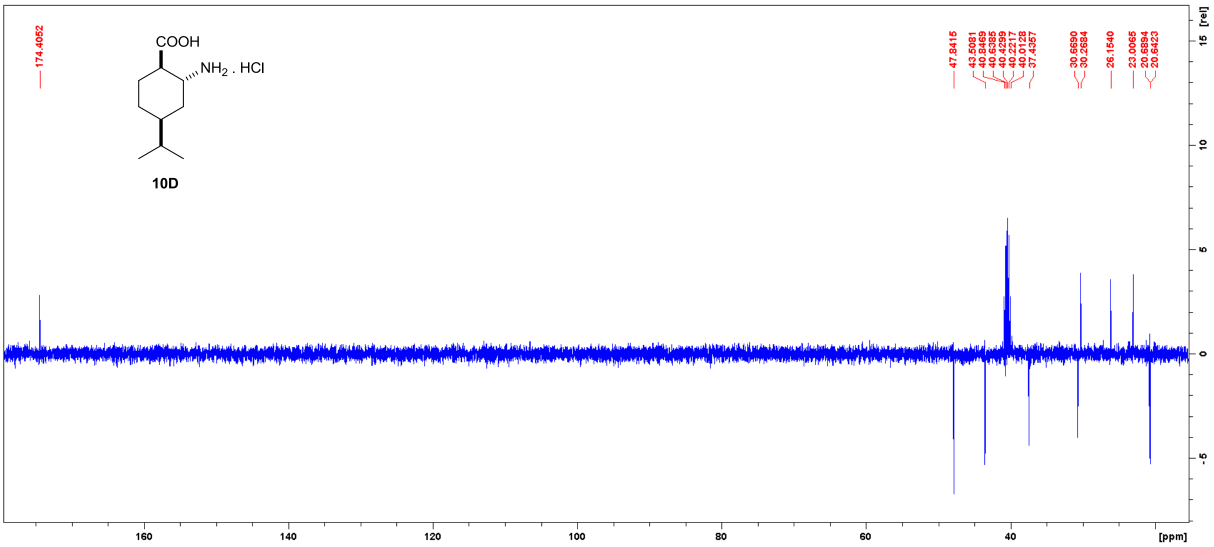

^{1}H and ^{13}C NMR of **11** ($CDCl_3$)

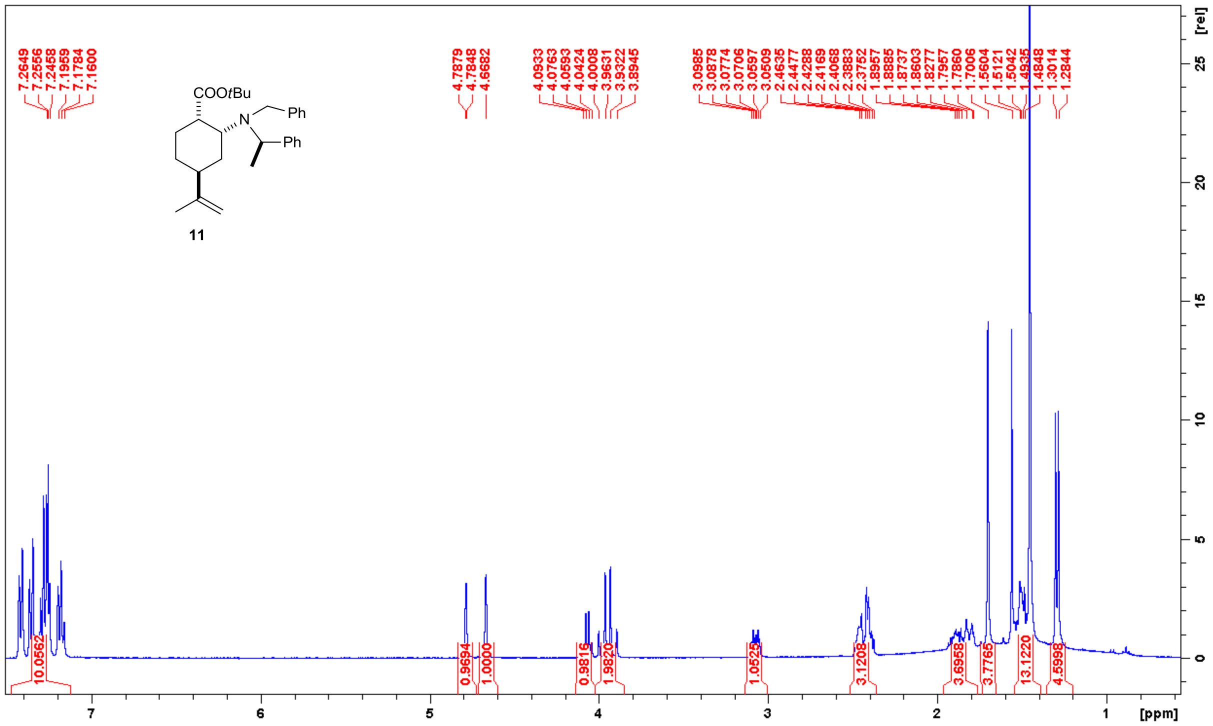

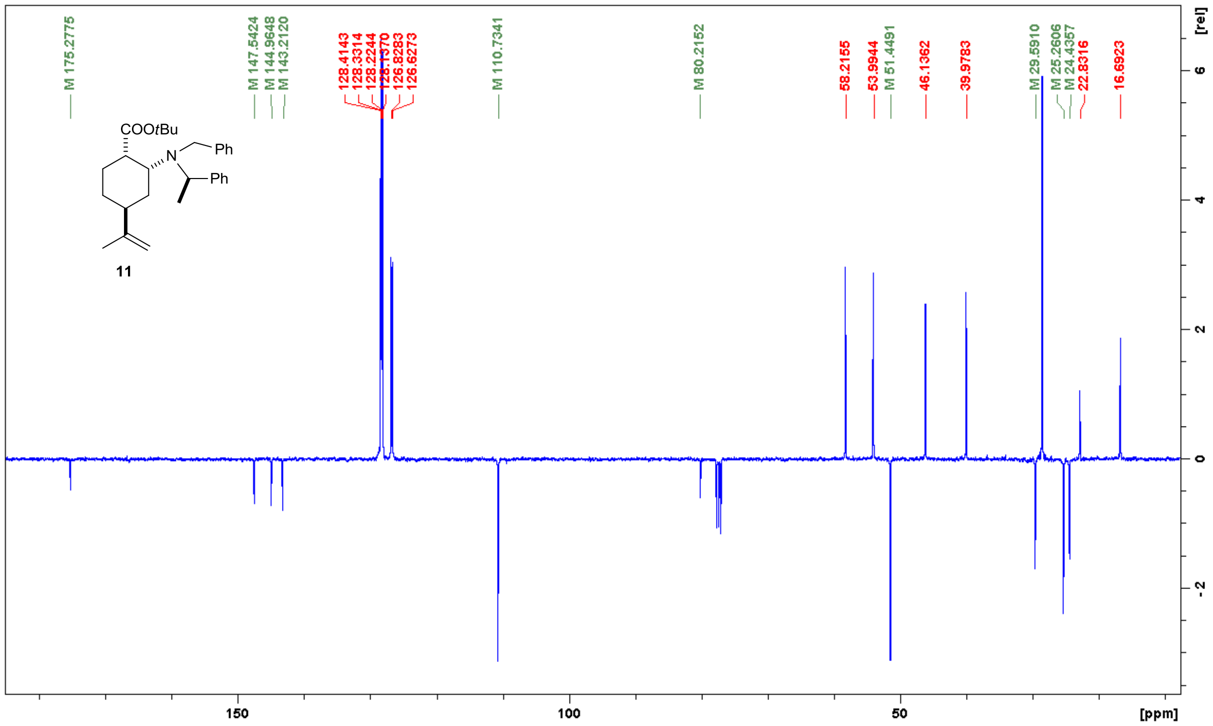

^{1}H and ^{13}C NMR of **12** ($CDCl_3$)

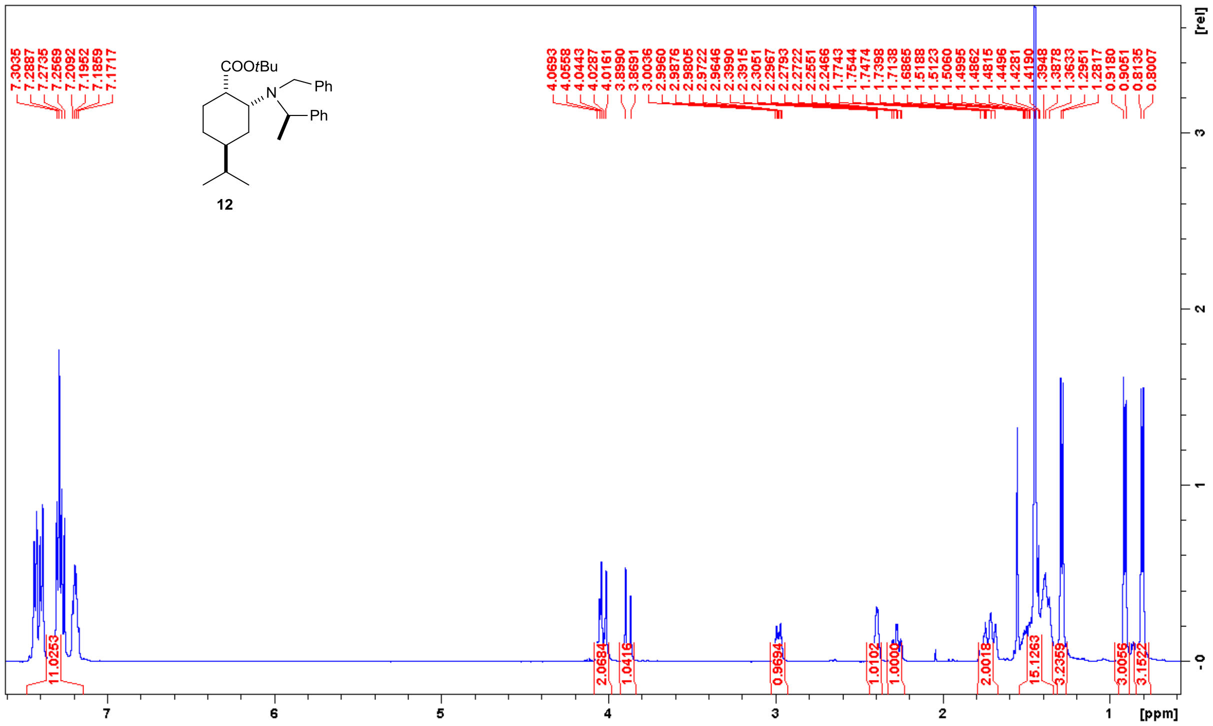

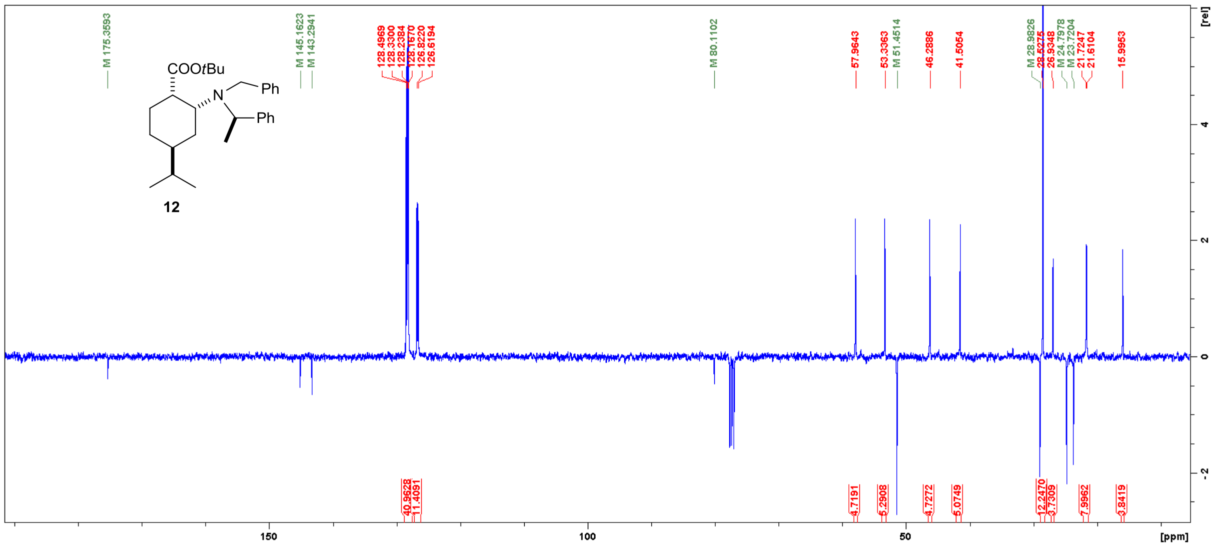

^{1}H and ^{13}C NMR of **13** ($CDCl_3$)

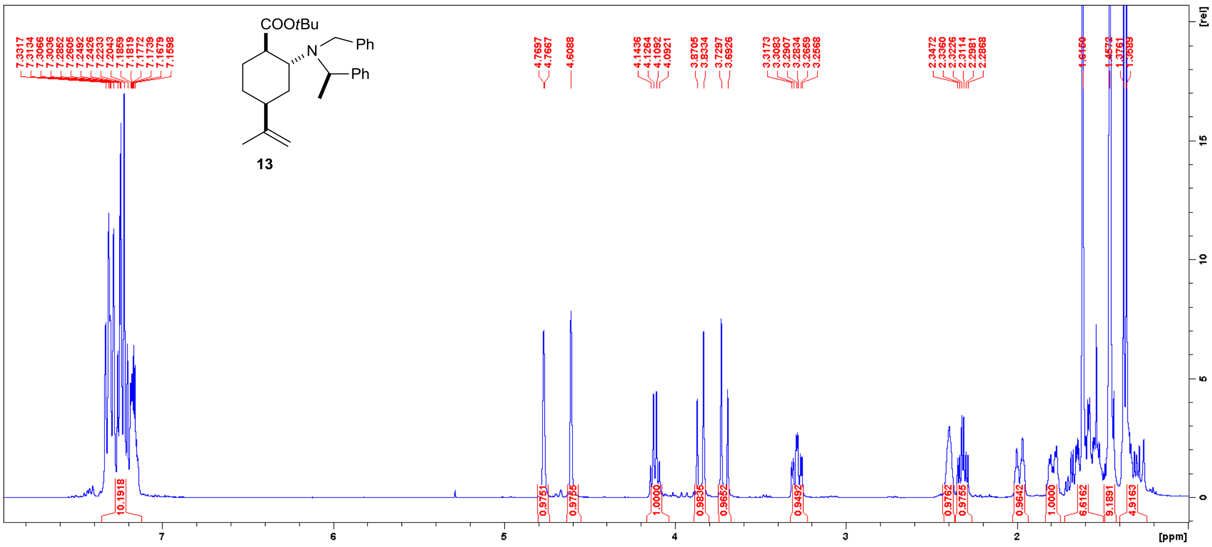

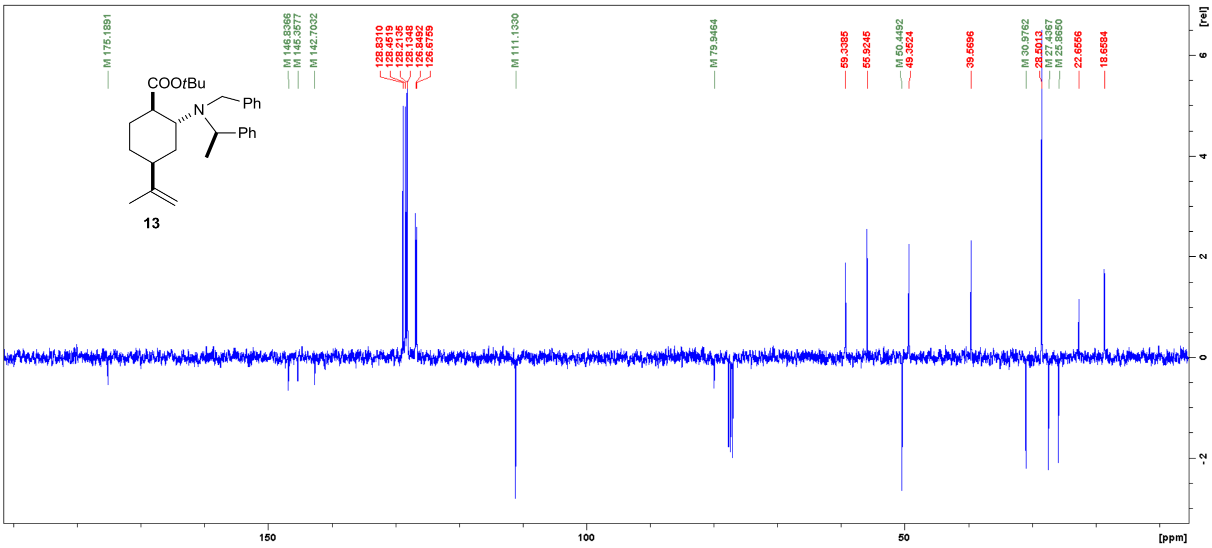

^{1}H and ^{13}C NMR of **14** ($CDCl_3$)

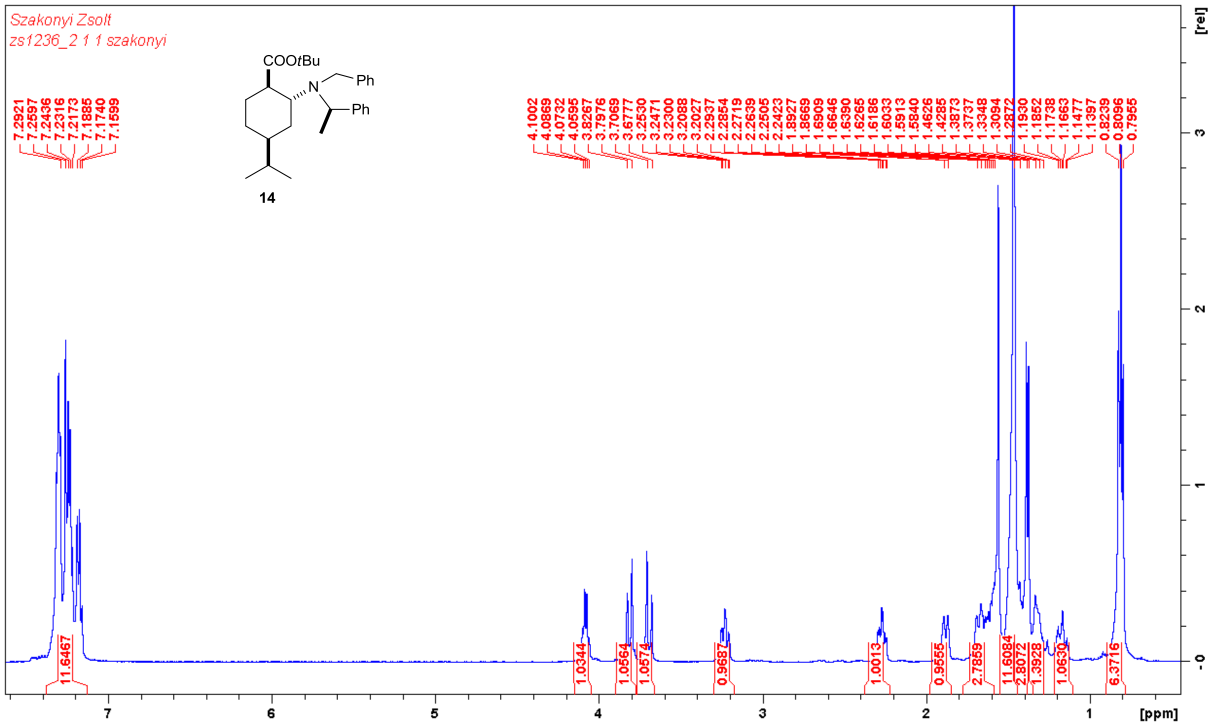

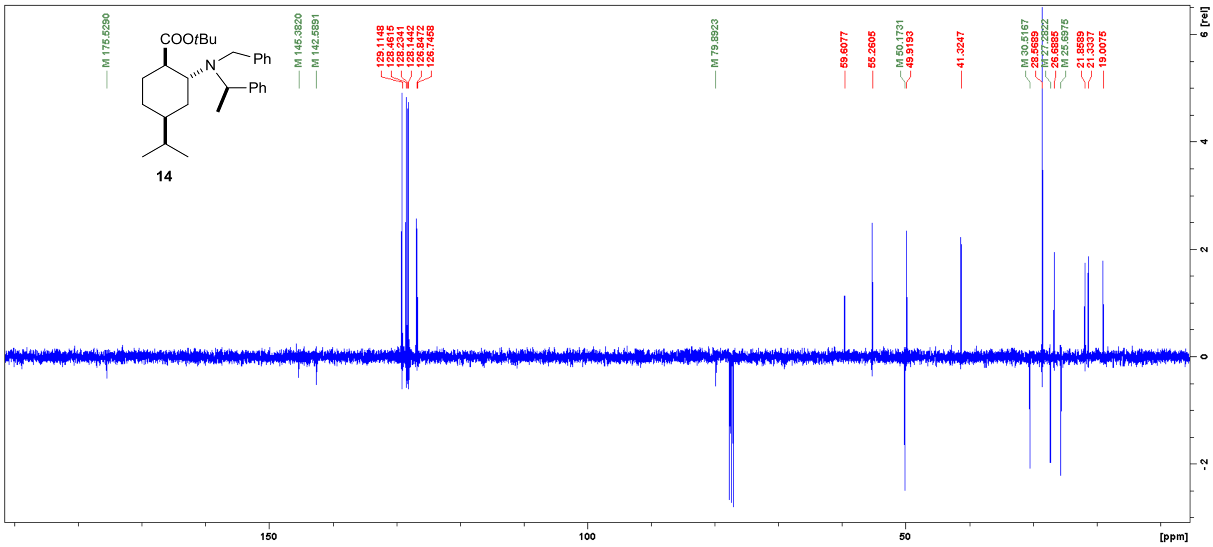

Supplement: File 1 — General information, experimental details, characterization data and copies of 1H and 13C NMR spectra. [file Beilstein_J_Org_Chem-10-2738-s001.pdf]
